# PLP Homeostasis Protein Is Required for Efficient Maturation of Aromatic Amino Acid Aminotransferase in *Salmonella enterica*

**DOI:** 10.1111/mmi.70072

**Published:** 2026-04-27

**Authors:** Brandi A. Buckner, Diana M. Downs

**Affiliations:** ^1^ Department of Microbiology University of Georgia Athens Georgia USA

**Keywords:** PLP homeostasis protein (PLPHP), pyridoxal‐5′‐phosphate (PLP), pyridoxamine, transamination, vitamin B_6_, YggS

## Abstract

Pyridoxal 5′‐phosphate (PLP) is an essential cofactor required for metabolic functions including amino acid biosynthesis. While the pathways of PLP synthesis are well characterized, it is unclear how newly synthesized PLP is delivered to apo PLP‐dependent enzymes (PLP‐DEs) in the cell. The highly conserved PLP Homeostasis Protein (PLPHP) is hypothesized to act as a PLP carrier that traffics the cofactor to PLP‐DEs. Prior studies have shown transfer of PLP between PLPHP homologs and PLP‐DEs in vitro. Data herein support a role for this transfer in vivo. This study utilizes a *Salmonella enterica* mutant strain with a synthetic requirement for PLPHP homolog, YggS. Genetic and biochemical analyses suggest that the synthetic phenotype of a *yggS aspC* mutant results from inefficient maturation of the aromatic amino acid aminotransferase, TyrB. This requirement for YggS can be bypassed with accumulation of B_6_ vitamer PMP, or upregulation of pyridoxal kinase (PdxK). Using TyrB as a target, we provide the first demonstration that YggS of *S. enterica* can mediate transfer of PLP to a PLP‐DE in vitro. In total, this study defines multiple in vivo maturation pathways for TyrB and provides a working model for the role of YggS in the maturation of TyrB.

## Graphical abstract

Cellular pool of aromatic amino acid aminotransferase proteins represented as holo‐ or apo‐ enzymes, reflecting presence or absence of associated PLP. Status of PLPHP is implicated in determining the ratio of holo‐ and apo‐TyrB.

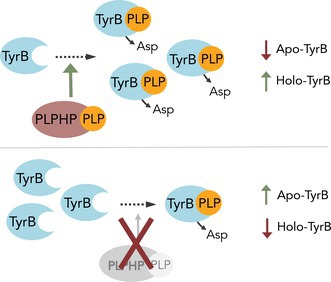

## 1 Introduction

Bacterial metabolism encompasses a complex set of reactions that involve enzymes, metabolites, and cofactors. The cofactor pyridoxal 5′‐phosphate (PLP) is the active form of vitamin B_6_ and is required for numerous metabolic reactions, including the biosynthesis of amino acids and secondary metabolites (Percudani and Peracchi 2009; Percudani and Peracchi 2003). PLP is required by all organisms and must be obtained either by *de novo* synthesis or salvage of one of the six B_6_ vitamers.

**FIGURE 1:**
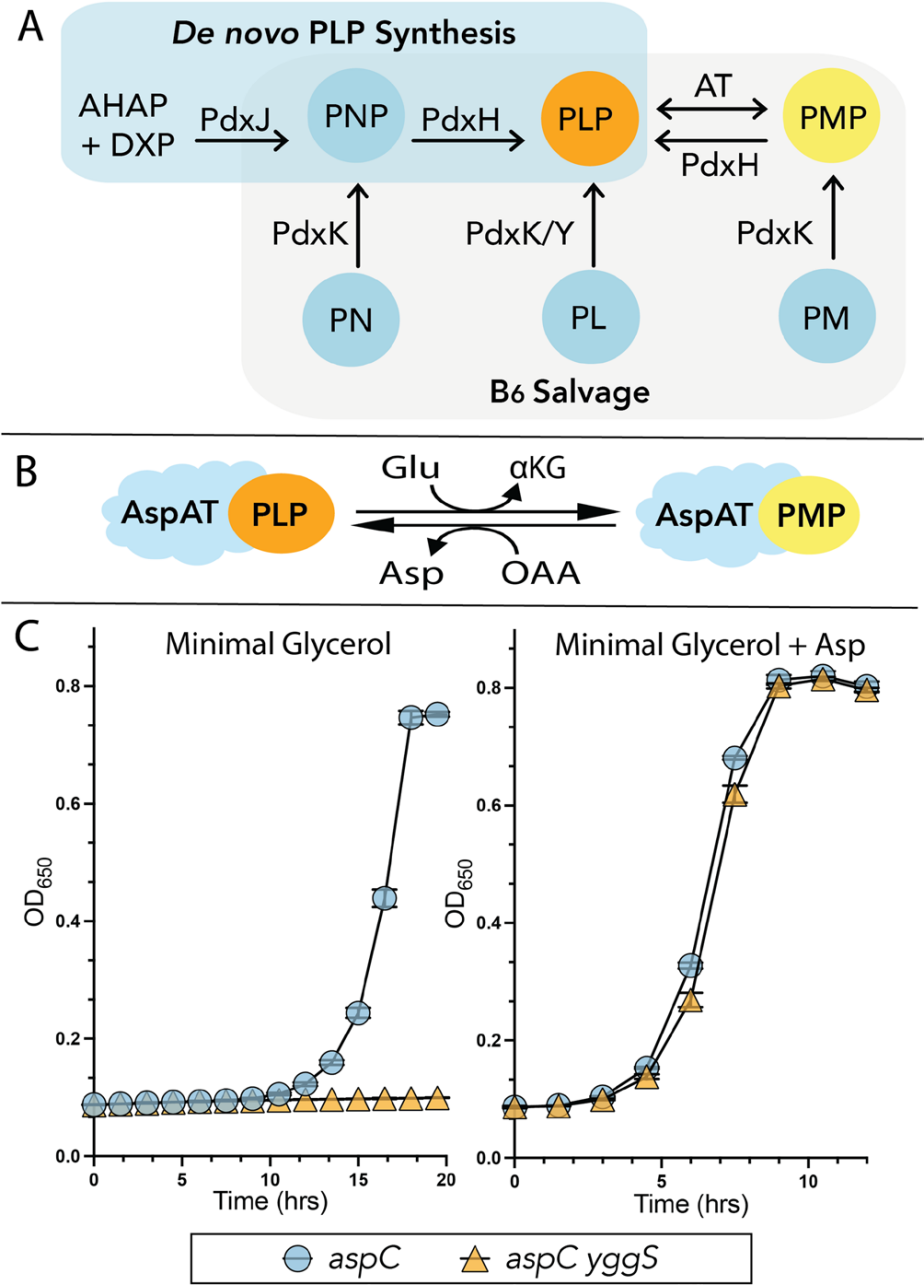
(A) Pathways for biosynthesis of pyridoxal 5′‐phosphate in *S. enterica* . The *de novo* biosynthesis pathway and the salvage pathway are highlighted in blue or grey boxes, respectively. B_6_ vitamers are represented by circles; vitamers that can act as cofactors are shown in orange (PLP) or yellow (PMP). PN(P), pyridoxine (5′‐phosphate); PL(P), pyridoxal (5′‐phosphate); PM(P), pyridoxamine (5′‐phosphate); AT, aminotransferases; AHAP, 3‐amino‐1‐hydroxyacetone 1‐phosphate; DXP, 1‐deoxy‐D‐xylulose 5‐phosphate. (B) Aspartate production by PLP‐dependent aspartate aminotransferases. The aspartate aminotransferase (AspAT) reaction consists of two half‐reactions: (i) the PLP‐bound enzyme accepts an amino group from glutamate to produce PMP (ii) the amine group of PMP is transferred to oxaloacetate (OAA); this results in the regeneration of PLP and produces aspartate. (C) Loss of YggS in an *aspC* background results in aspartate auxotrophy. Growth of *aspC* (circles, DM16409) and *aspC yggS* (triangles, DM16841) *S. enterica* strains in minimal glycerol media in the absence or presence of 1.8 mM aspartate. Error bars represent the standard deviation of three biological replicates.

Most, if not all, organisms can obtain PLP by salvaging B_6_ vitamers from the environment. The B_6_ salvage pathway is largely conserved across the domains of life. In general, pyridoxal kinase (PdxK, EC 2.7.1.35) phosphorylates the vitamers pyridoxine (PN), pyridoxamine (PM), and pyridoxal (PL). In this process, PLP is generated either directly or via oxidation of PMP/PNP vitamers by the PNP/PMP oxidase, PdxH (EC 1.4.3.5) (Figure 1A). PLP is commonly referred to as the biologically active form of B_6_, but PMP can also serve a catalytic role in aminotransferases. During catalysis, aminotransferases interconvert PMP and PLP (Figure 1B); this activity can compensate in vivo for the PMP oxidase activity of PdxH, allowing PM to serve as the sole source of B_6_ in the absence of PdxH (Chen et al. 2019; Ito et al. 2022; Lam and Winkler 1992; Vu and Downs 2021a). While some organisms also encode a pyridoxal reductase (EC 1.1.1.65) that converts PL to PN, *Salmonella enterica* does not encode this activity (Ito and Downs 2020).

In addition to salvage, many microbes and plants have the capacity to synthesize PLP *de novo* (Figure 1A). In most organisms that rely on *de novo* synthesis, PLP results from the condensation of glyceraldehyde‐3‐phosphate, ribose‐5‐phosphate, and ammonium by a PLP synthase (EC 4.3.3.6). However, in some gamma‐proteobacteria like *Escherichia coli* and *Salmonella enterica* , PLP is synthesized through a deoxyylulose‐5‐phopshate (DXP)‐dependent pathway. In this pathway, DXP and 3‐phosphohydroxy‐1‐aminoacetone are condensed by PNP synthase (PdxJ, EC 2.6.99.2) to generate PNP, which is oxidized by PdxH to generate PLP (Figure 1A).

Although vital to the cell, PLP is a damage‐prone aldehyde that readily reacts with free amine groups (Lerma‐Ortiz et al. 2016). The reactivity of PLP led to the prediction that B_6_ vitamer homeostasis is carefully maintained to minimize free PLP in the cell (di Salvo et al. 2011). B_6_ homeostasis is thought to be maintained by several mechanisms including transcriptional regulation, dephosphorylation of free PLP by phosphatases, and feedback‐inhibition of PLP synthesizing enzymes (di Salvo et al. 2011; Moccand et al. 2011; Tramonti et al. 2021). The latter mechanism is exemplified by PL kinase (PdxK) and PMP/PNP oxidase (PdxH), which are feedback inhibited by the PLP they produce (Barile et al. 2024, 2021, 2019; di Salvo et al. 2015; Ghatge et al. 2012; Musayev et al. 2003; Yang and Schirch 2000; Zhao and Winkler 1995). In vitro, PLP‐bound PdxK and PdxH can transfer PLP to the few apo PLP‐dependent enzymes (PLP‐DEs) tested (Al Mughram et al. 2022; Cheung et al. 2003; Deka et al. 2019; Ghatge et al. 2016, 2012; Kim et al. 1988; Yang and Schirch 2000). This transfer restores the activity of the donor enzyme and activates the target PLP‐DE. The PdxK and PdxH enzymes are presumed to function as delivery systems in vivo to minimize the release of free PLP into the cellular milieu. Despite abundant biochemical evidence for PLP transfer by PdxH and PdxK in vitro, whether these exchanges contribute to the in vivo maturation of PLP‐DEs remains unclear.

In the last decade, Pyridoxal 5′‐Phosphate Homeostasis Proteins (PLPHP) have been implicated in B_6_ homeostasis. PLPHPs bind PLP through a Schiff base linkage. In microbes, plants, zebrafish, and humans, loss of PLPHP activity results in disruption of vitamin B_6_ homeostasis and alterations in amino acid pools (Ciapaite et al. 2023; Darin et al. 2016; Farkas and Fitzpatrick 2024; Ito et al. 2019; Johnstone et al. 2019; Vu et al. 2020). Some mutations in human PLPHP result in a potentially lethal epilepsy that responds to vitamin B_6_ supplementation (Darin et al. 2016; Johnstone et al. 2019). Biochemical experiments show that PLPHP from *Escherichia coli* and *Arabidopsis thaliana* can transfer PLP in vitro to serine hydroxymethyltransferase and D‐amino acid aminotransferase, respectively (Farkas and Fitzpatrick 2024; Tramonti et al. 2022). Based on these in vitro results and the B_6_‐responsive epilepsy associated with PLPHP variants, it is plausible that PLPHP proteins have a role in PLP delivery.

In otherwise wildtype *S. enterica* and *E. coli* strains, loss of PLPHP does not generate an obvious growth defect, suggesting it is a metabolic modulator rather than an essential protein. Subtle effects caused by loss of a protein can often be enhanced by changing genetic background or growth conditions to generate phenotypes that can be exploited genetically (Downs et al. 2018; Enos‐Berlage et al. 1998; Fulton and Downs 2022; Kim et al. 2010; Martinez‐Gomez et al. 2004; Paxhia and Downs 2022). In *S. enterica* , a mutant lacking both the PLPHP homolog, YggS, and the aspartate aminotransferase, AspC (EC 2.6.1.1), fails to grow in minimal glycerol media without aspartate (Figure 1C) (Vu and Downs 2021b). Importantly, strains lacking either protein alone grow well in the absence of aspartate.

In *S. enterica* , two PLP‐dependent aspartate aminotransferases (AspATs) can fulfill the cellular aspartate requirement: aspartate aminotransferase (AspC, EC 2.6.1.1) and aromatic amino acid aminotransferase (TyrB, EC 2.6.1.57) (Figure 1B). Although TyrB has low AspAT activity compared to AspC, it has enough activity to compensate for loss of *aspC* under certain conditions (Gelfand and Steinberg 1977; Powell and Morrison 1978). Significant data, including those presented here, show that TyrB‐dependent aspartate biosynthesis is deficient in the absence of YggS (Vu and Downs 2021b). This study took advantage of the synthetic aspartate auxotrophy of an *aspC yggS* mutant to gain insights into the function of YggS. The data support a working model in which YggS facilitates the maturation of TyrB with PLP. Insights derived from this work provide a framework for continuing efforts to dissect the in vivo interactions between PLPHP and PLP‐DEs.

## 2 Results and Discussion

### 2.1 The Aspartate Requirement of an *aspC yggS* Mutant Is Not Caused by PNP, Lack of Substrates, or Repression of *tyrB* Gene Expression

**FIGURE 2:**
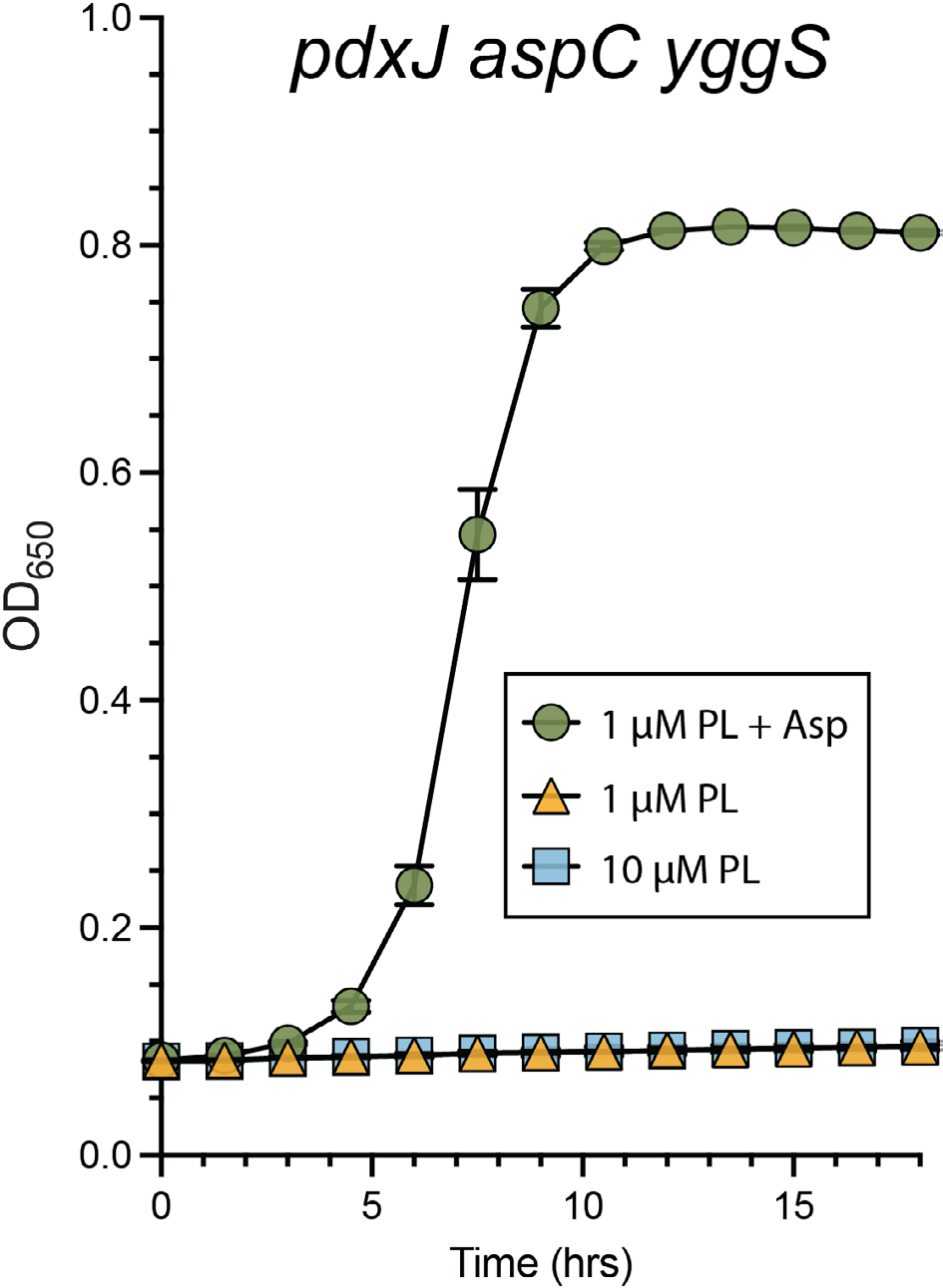
PNP is not responsible for the YggS‐dependent growth phenotype. Growth of *pdxJ aspC yggS* strain (DM16703) in minimal glycerol media supplemented with 10 μM PL (squares), 1 μM PL (triangles), or 1 μM PL and 1.8 mM aspartate (circles). Error bars represent the standard deviation of three biological replicates.

The aspartate requirement of an *aspC yggS* mutant is eliminated upon TyrB overexpression, indicating that TyrB activity is limiting in this system (Vu and Downs 2021b). It was previously suggested that the aspartate requirement of an *aspC yggS* mutant may be due to inhibition of TyrB by PNP, which accumulates in a *yggS* mutant (Vu and Downs 2021b). To address this possibility, endogenous PNP synthesis was blocked by disrupting the *pdxJ* gene in *aspC* and *aspC yggS* genetic backgrounds. PL, a vitamer that is assimilated without a PN(P) intermediate, was provided (Figure 2). If the growth defect observed in an *aspC yggS* mutant is due to PNP, eliminating all exogenous and endogenous sources of PNP would nullify the effect of the *yggS* mutation. Instead, the *pdxJ aspC yggS* triple mutant retains an aspartate requirement in the presence of PL (Figure 2). A similar result was obtained when a different gene in the PNP synthesis pathway (*pdxB*) was disrupted (Figure S1). Thus, the *yggS* phenotype is not eliminated by blocking PNP synthesis. In total, these results support the conclusion that inhibition of TyrB by PNP is not directly responsible for the aspartate auxotrophy of an *aspC yggS* mutant.

Loss of YggS does not increase the aspartate requirement of an *aspC* strain (Vu and Downs 2021b). It was formally possible that loss of YggS prevents sufficient aspartate biosynthesis by decreasing the expression of *tyrB*. RT‐qPCR analysis found no significant difference in expression of *tyrB* between *aspC* and *aspC yggS* mutants grown in minimal medium with aspartate (Table S1). While not definitive, these data make the possibility of a transcriptional difference caused by lack of *yggS* unlikely. Consistently, there is no significant difference in in vitro AspAT activity in the crude extracts of *aspC* and *aspC yggS* strains (Vu and Downs 2021b). Finally, it was possible that loss of YggS limits the AspAT substrates: glutamate and oxaloacetate. When provided at 1.8 mM, neither glutamate or oxaloacetate (individually or in combination) allow growth of an *aspC yggS* mutant in the absence of aspartate (Figure S2), indicating that AspAT substrates are not limited by the loss of YggS.

The above data highlight a challenge in probing the cellular role of YggS. Overwhelming evidence, presented herein and elsewhere (Vu and Downs 2021b), indicates that in the absence of YggS, there is a reduction in TyrB activity in vivo. This difference in AspAT activity has not been recapitulated with the conditions utilized in crude extract assays. Despite caveats with each approach, the data in total suggest that *aspC yggS* mutants have a post‐transcriptional defect in TyrB‐dependent aspartate biosynthesis that has not been captured in vitro. We hypothesize that YggS impacts the maturation of TyrB, a role consistent with reports that some PLPHP homologs transfer PLP to model apo‐enzymes in vitro (Farkas and Fitzpatrick 2024; Tramonti et al. 2022). While we do not see YggS‐dependent changes in TyrB activity in vitro, crude extracts do not accurately reflect the intracellular environment (Kontopidis and Patergiannakis 2025). For instance, since PLP is a highly reactive molecule, the kinetics of PLP binding to apo‐enzymes is expected to be affected by changes to biochemical conditions such as molecular crowding and spaciotemporal organization. Prompted by the caveats of biochemical analysis using crude extracts, we used in vivo genetic analyses to explore the mechanism of TyrB maturation.

### 2.2 TyrB‐Dependent Aspartate Synthesis Is Vulnerable to B_6_ Vitamer Availability

**FIGURE 3:**
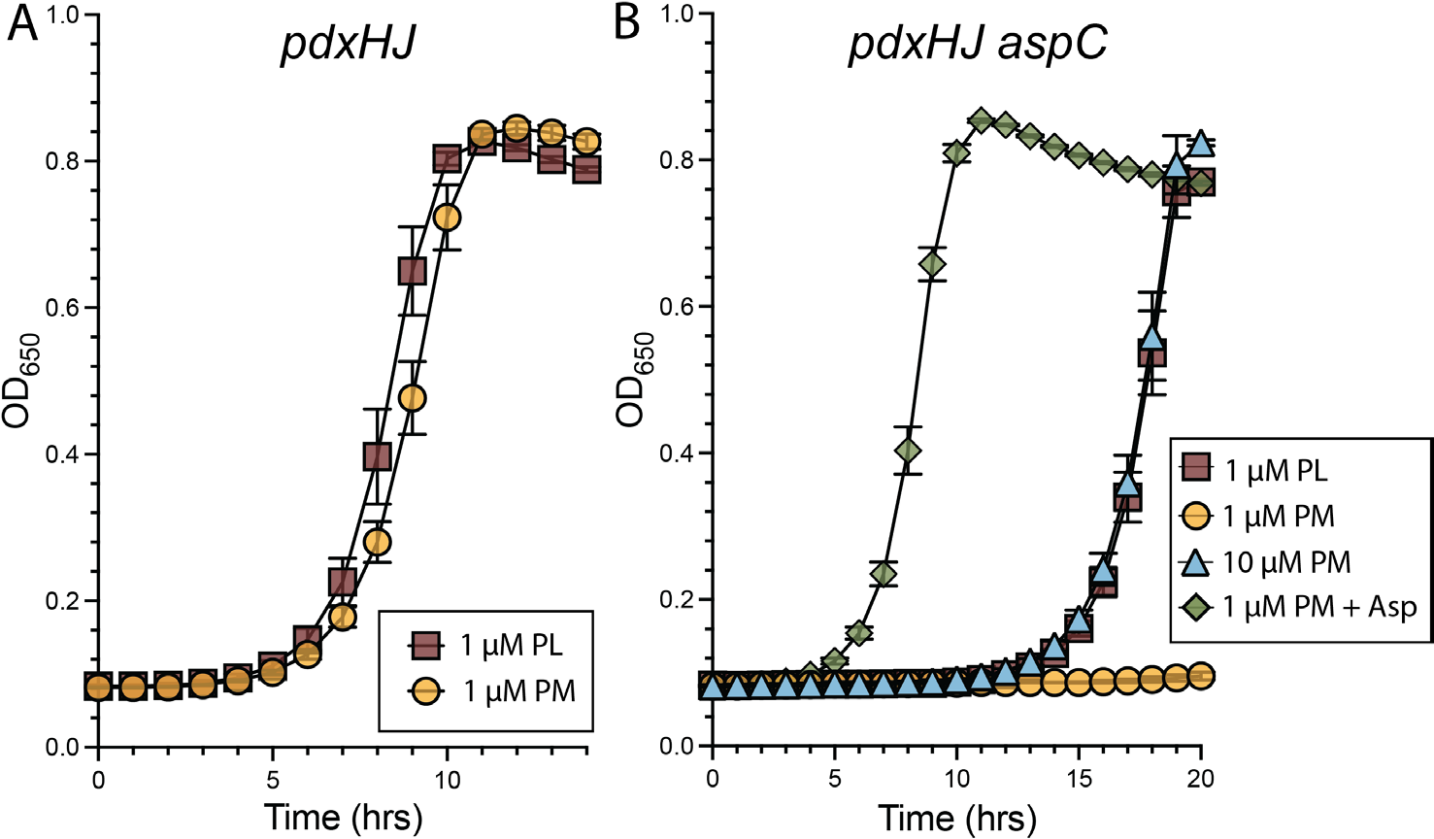
Mutants lacking AspC require higher PM to satisfy a B_6_ auxotrophy. (A) Growth of *pdxHJ* strain (DM16397) in minimal glycerol media supplemented with 1 μM PL (squares) or PM (circles). (B) Growth of *pdxHJ aspC* strain (DM16704) in minimal glycerol media with 1 μM PL (squares), 1 μM PM (circles), 10 μM PM (triangles), or 1 μM PM with 1.8 mM aspartate (diamonds). Error bars represent the standard deviation of three biological replicates.

Strains lacking both *pdxJ* and *pdxH* were utilized to eliminate *de novo* PLP synthesis and allow analysis of individual B_6_ vitamer salvage pathways. The B_6_ requirement of strains lacking *de novo* PLP synthesis (*pdxJH*) can be satisfied with 1 μM PL or PM (Figure 3A). When TyrB‐dependent aspartate synthesis is required for growth (i.e., in an *aspC* mutant), the profile of the vitamer requirement changes. A *pdxJH aspC* mutant grows with 1 μM PL but requires 10 times more PM than the *pdxJH* mutant (Figure 3B). Importantly, in the presence of aspartate, the PM requirement of the *pdxHJ aspC* mutant returns to 1 μM. This result emphasizes that TyrB‐dependent aspartate formation specifically requires 10 μM PM while 1 μM PM is sufficient for the required activity of all other PLP‐DEs.

These data suggest that TyrB is loaded less efficiently with cofactor when salvaging PM as opposed to PL. Compared to AspC, TyrB is a low abundance enzyme and generates aspartate 100‐fold less efficiently (Huang et al. 2023; Powell and Morrison 1978). Inefficient cofactor loading of TyrB could result in even less AspAT activity, which would prevent generation of sufficient aspartate for growth in the absence of AspC. The stringency of B_6_ vitamer requirements during TyrB‐dependent aspartate synthesis suggested that growth of an *aspC* mutant could provide a sensitive system to probe the role of YggS in B_6_ utilization.

### 2.3 A Working Model Implicates YggS in the Maturation of TyrB

**FIGURE 4:**
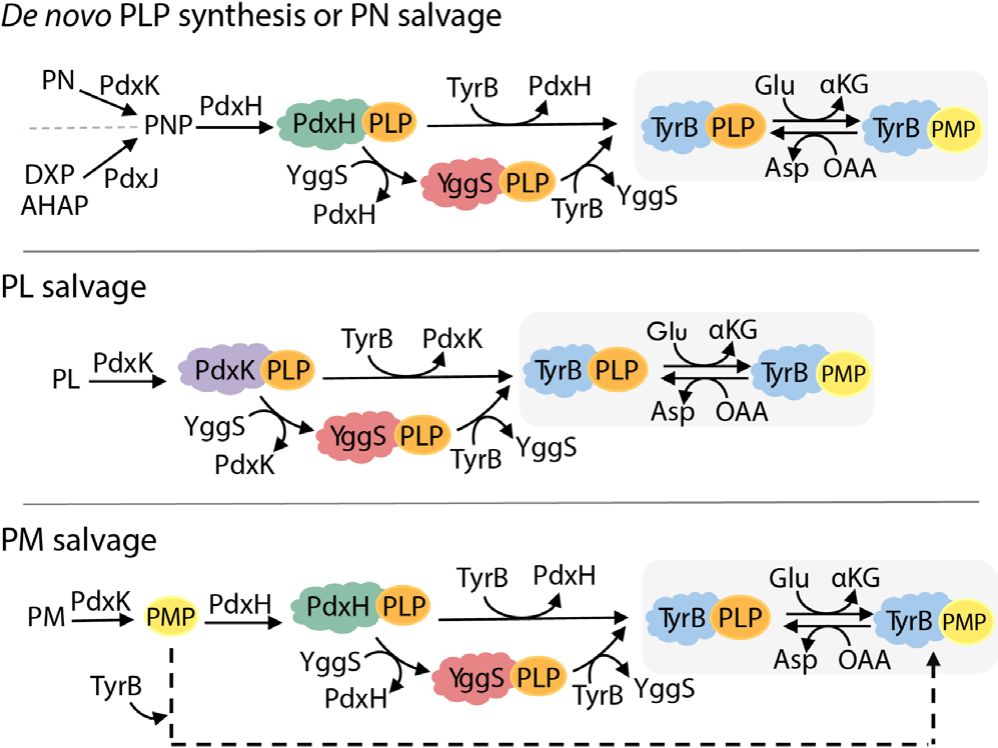
Working model of YggS‐mediated maturation of TyrB. A working model for the role of YggS in trafficking PLP is schematically shown and described in the text. YggS acts as an intermediate PLP carrier between PdxH or PdxK and TyrB. (Top panel) During PN salvage or *de novo* PLP synthesis, TyrB can be matured by PdxH●PLP with low efficiency or YggS●PLP with high efficiency. (Middle panel) During PL salvage, TyrB can be matured by PdxK●PLP with low efficiency or YggS●PLP with high efficiency. (Bottom panel) During PM salvage, TyrB can be loaded with cofactor by PdxK●PLP with low efficiency or YggS●PLP with high efficiency. Upon accumulation, free PMP can also efficiently mature TyrB.

In the following sections, we systematically report the logic leading to and the data resulting from growth analyses that provide a proxy for TyrB maturation. In total, results obtained and interpreted below, in combination with studies described in the literature, support the working model shown in Figure 4. To facilitate evaluation, the key observations from growth analyses that led to this model are summarized in Table S2 and presented more fully in the text. The working model has four main components:

1. PLP can be delivered to TyrB by PdxH, PdxK or YggS.
2. Efficient transfer of PLP from PdxH to TyrB requires YggS. Efficiency is measured as the ability of TyrB to satisfy the aspartate requirement of the cell.
3. Efficient transfer of PLP from PdxK to TyrB requires YggS. This requirement can be overcome with increased [PdxK].
4. Maturation of TyrB with PMP is independent of YggS. The high [PMP] required for TyrB maturation can be generated by PM supplementation if either *pdxH* is blocked or [PdxK] is increased.

### 2.4 YggS Facilitates Maturation of TyrB With PLP

*pdxHJ* and *pdxHJ yggS* mutants grow equally well when 1 μM PL or PM are provided as the source of B_6_ (Figure S3). This result shows that the requirement for PLP/PMP is not significantly altered by the loss of YggS and that 1 μM vitamer results in sufficient activity of cellular PLP‐DEs for full growth.

**FIGURE 5:**
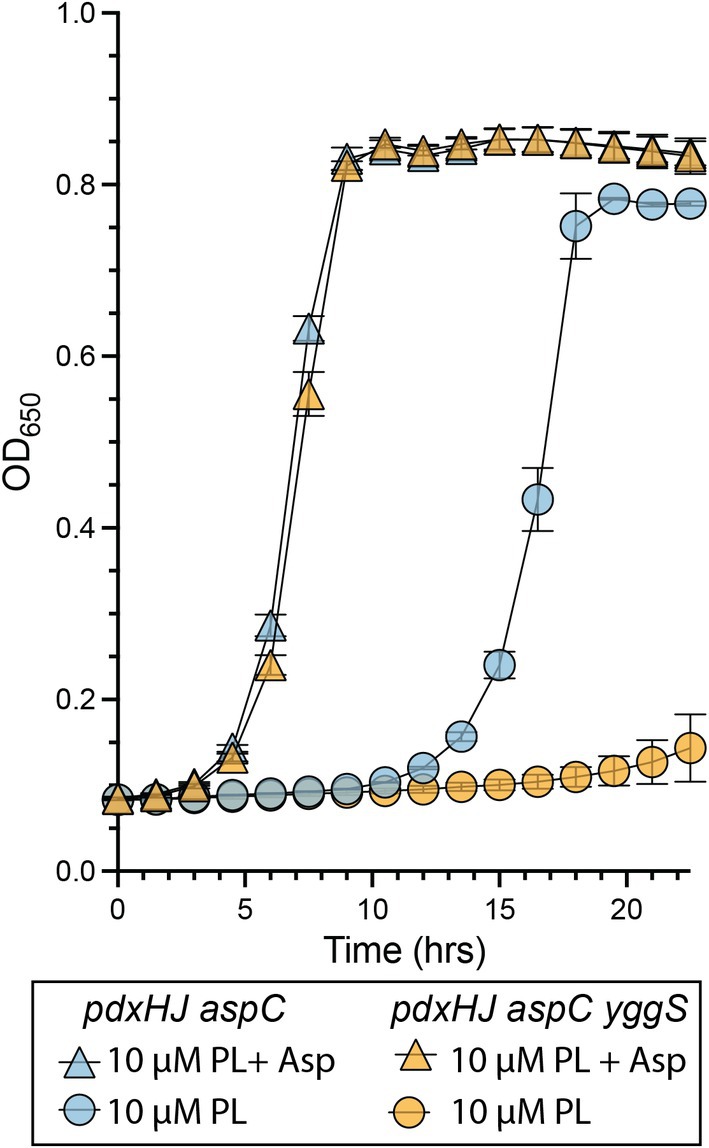
YggS‐dependent aspartate requirement is not eliminated by exogenous pyridoxal. Growth of *pdxHJ aspC* (blue symbols, DM16704) and *pdxHJ aspC yggS* (yellow symbols, DM16705) strains in minimal glycerol media with 10 μM PL (circles) or 10 μM PL with 1.8 mM aspartate (triangles). Error bars represent the standard deviation of three biological replicates.

When a cell relies on TyrB‐dependent aspartate synthesis for growth and lacks YggS, the requirements for vitamer salvage change. A *pdxJH aspC yggS* mutant does not grow on minimal medium supplemented with PL, whether it is provided at 1 or 10 μM (Figure 5, data not shown for 1 μM PL). This result was striking since the parental *pdxJH aspC* strain grows with the addition of 1 or 10 μM PL. Thus, when cell growth depends on TyrB‐dependent aspartate synthesis, YggS is required if PL is provided as the sole source of B_6_.

**FIGURE 6:**
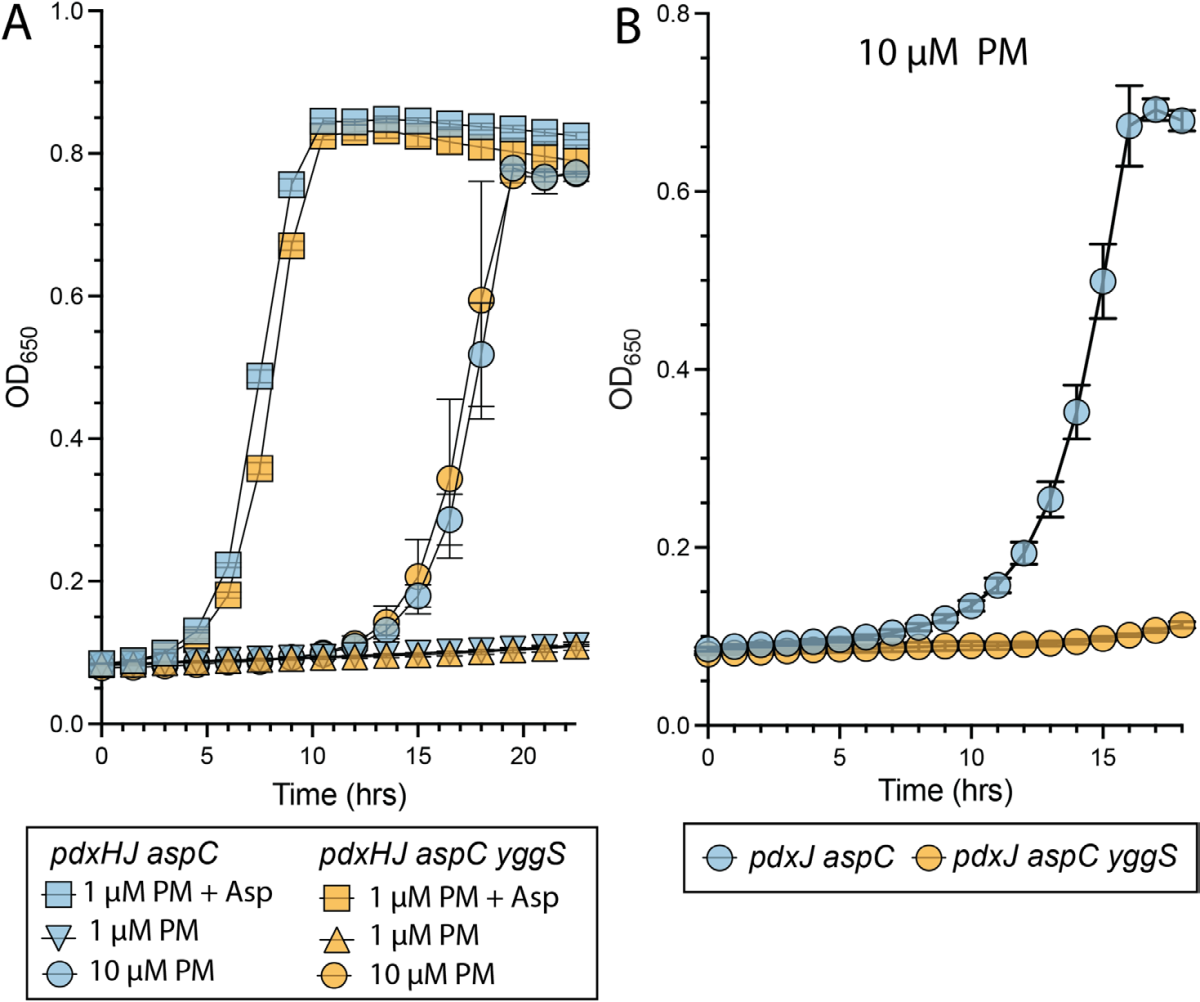
PdxH activity determines if PM salvage rescues YggS‐dependent aspartate auxotrophy. (A) Growth of *pdxHJ aspC* (blue symbols, DM16704) and *pdxHJ aspC yggS* (yellow symbols, DM16705) in minimal glycerol media supplemented with 10 μM PM (circles), 1 μM PM (triangles), or 1 μM PM with 1.8 mM aspartate (squares). Error bars represent the standard deviation of three biological replicates. (B) Growth of *pdxJ aspC* (blue, DM16702) and *pdxJ aspC yggS* (yellow, DM16703) mutant strains on minimal media glycerol supplemented with 10 μM PM.

In contrast, salvage of PM in the *pdxHJ aspC* background does not require YggS. Like the parental strain (*pdxHJ aspC*), a *pdxHJ aspC yggS* mutant requires 10 μM PM for efficient TyrB‐dependent aspartate synthesis (Figure 6A). However, in the presence of functional PdxH, efficient TyrB‐dependent aspartate synthesis during PM salvage becomes dependent on YggS (Figure 6B). The YggS‐independent maturation of TyrB during PM salvage requires 10 μM PM and loss of PdxH activity. This result suggests that YggS‐independent TyrB activation requires PMP accumulation (see Figure 4). PMP accumulation could promote TyrB●PMP formation, while the PMP oxidase activity of PdxH would prevent the accumulation of PMP needed to efficiently activate TyrB. A PMP carrier has not been described, suggesting that maturation of TyrB (and other aminotransferases) with PMP occurs via diffusion in vivo. This scenario is supported by in vitro experiments showing that apo‐aminotransferases can be reconstituted with free PMP (Tobler et al. 1986).

In total, these analyses show that YggS is required when TyrB must be activated by PLP produced by PdxH or ‐K (see Figure 4). YggS is required during PM salvage only when PdxH is available to generate PLP. YggS is not required if an efficient PMP‐dependent maturation pathway is available due to the elimination of PdxH. These data support a scenario where YggS is specifically required for maturation of TyrB with the vitamer PLP. Controls show that derepression of *tyrB* (via a *tyrR* mutation) (Figure S4) or aspartate supplementation negates the effect of a *yggS* mutation in the *aspC* mutant background. These data emphasize that growth of an *aspC yggS* mutant is limited only by insufficient TyrB‐dependent aspartate synthesis.

### 2.5 YggS Transfers PLP to TyrB In Vitro

In total, genetic analyses suggested that YggS could facilitate the activation of TyrB activity with PLP, but not PMP. Considering that YggS homologs from *E. coli* and *A. thaliana* traffic PLP to PLP‐DEs in vitro (Farkas and Fitzpatrick 2024; Tramonti et al. 2022), we hypothesized that YggS of *S. enterica* impacts TyrB activity in vivo by delivering PLP to the apo‐aminotransferase.

To test the plausibility of our hypothesis, transfer of PLP from YggS to TyrB was assayed in vitro. Apo‐TyrB was generated by treating the purified protein with cysteine. Cysteine reacts with free‐ and protein‐bound PLP to form a diffusible adduct that can be removed by buffer exchange, making it effective in the preparation of apo‐PLP‐DEs (Schonbeck et al. 1975; Tramonti et al. 2022). The apo‐TyrB preparation had little AspAT activity, confirming the successful removal of cofactor. Addition of 10‐fold excess PLP to apo‐TyrB increased AspAT activity to a level presumed to reflect the activity displayed by holo‐TyrB (Table 1). Preincubation of apo‐TyrB with YggS generates ~5‐times more AspAT activity than apo‐TyrB alone. The YggS preparation alone does not have AspAT activity, prompting the conclusion that AspAT activity measured after the coincubation reflects PLP transfer from YggS to TyrB. No exogenous PLP was provided during overexpression or purification of YggS, effectively eliminating the possibility of free PLP in the YggS preparation activating TyrB. Spectral analyses indicate that ~38% of YggS in the protein preparation is bound to PLP (Table 2). This as‐purified YggS protein preparation restored 12% of the maximal AspAT activity of TyrB after coincubation.

**TABLE 1:** Incubation of apo‐TyrB with YggS increases AspAT activity. AspAT activity was assayed via a coupled enzyme assay as described in Materials and Methods. Apo‐TyrB (4 μM) preincubated with PLP (40 μM) was assayed to determine the activity of holo‐TyrB. Apo‐TyrB was preincubated (10 m) with YggS (40 μM) that was or was not treated with cysteine prior to assaying AspAT activity. Standard deviation of three technical replicates is presented. All differences in AspAT activity were significant (*p* < 0.01) as determined by ANOVA analysis followed by Tukey's multiple comparison test except Apo‐TyrB versus YggS with PLP.

Protein components	AspAT activity^a^	Percent activity
Holo‐TyrB^b^	208.5 ± 5.4	100%
Apo‐TyrB	4.8 ± 0.5	2.3%
Apo‐TyrB with YggS	25.0 ± 1.1	12.0%
Apo‐TyrB with cysteine‐treated YggS	15.5 ± 0.4	7.4%
YggS with free PLP	−0.1 ± 0.1	0.0%

^a^ AspAT activity is reported as mmol NADH oxidized min^−1^.

^b^ Holo‐TyrB is the preparation of apo‐protein to which PLP (40 μM) was added 10 min prior to assay initiation.

**TABLE 2:** Percentage of holo‐YggS in protein preparations. 150 μM of the indicated YggS protein preparation was denatured in 0.1 M NaOH. The total concentration of PLP (μM) in each sample was calculated using the absorbance at 388 nm and the extinction coefficient of PLP in 0.1 M NaOH (ε_340_ = 6600 M^−1^ cm^−1^) (Peterson and Sober 1954). PLP concentration was calculated using three technical replicates and is reported with standard deviation. The percentage of holo‐YggS in each protein preparation was calculated by assuming all PLP was protein‐bound prior to denaturing.

Protein (150 μM)	Total [PLP] (μM)	*p*	Percent Holo‐YggS
YggS (as purified)	57.6 ± 1.8	0.0140	38%
Cysteine‐treated YggS	51.9 ± 1.2		35%

Cysteine treatment of the *E. coli* protein results in a preparation of largely apo‐YggS (Tramonti et al. 2022). Unexpectedly, cysteine treatment of the *Salmonella* protein (SeYggS) failed to significantly remove PLP and only reduced the amount of holo‐YggS by 3% (Table 2). The recalcitrance of *S. enterica* YggS to PLP removal by cysteine suggests either a unique feature of this protein or unidentified/subtle difference(s) in the experimental protocols and was not pursued here. Despite the small decrease in holo‐YggS after cysteine treatment, it is satisfying that slightly less AspAT activity is obtained when apo‐TyrB is incubated with the cysteine‐treated YggS preparation (Table 1). In total, the data are consistent with an in vitro transfer of PLP from YggS•PLP to TyrB and the possibility that YggS acts as a PLP carrier in vivo.

### 2.6 PdxK Can Suppress Defects in PLP Trafficking Caused by Loss of YggS

In vitro studies with pure components, including those herein, show that PdxK, PdxH, and YggS homologs can traffic PLP to some PLP‐DEs (Yang and Schirch 2000; Ghatge et al. 2012; Deka et al. 2019; Al Mughram et al. 2022; Tramonti et al. 2022; Farkas and Fitzpatrick 2024). However, data to support a role for the former two proteins in PLP delivery in vivo are lacking. The *aspC yggS* system was used to assess the ability of these putative PLP carriers to activate TyrB in vivo and thus facilitate sufficient TyrB‐dependent aspartate synthesis for growth.

In *S. enterica* , *pdxK* transcription increases by ~80‐fold in the absence of the transcriptional repressor, PtsJ (Tramonti et al. 2017). A lesion in *ptsJ* was introduced to *pdxHJ aspC* mutant strains to determine if increased [PdxK] affects TyrB activation during vitamer salvage. As noted previously, a *pdxJH aspC* mutant requires 10 μM PM to support sufficient TyrB‐dependent aspartate synthesis for growth (Figure 3). When PtsJ is absent (*pdxJH aspC ptsJ*), 1 μM PM is sufficient for growth, suggesting that increased [PdxK] eliminates the higher PM requirement conferred by the *aspC* mutation (Figure S5). These data support a simple scenario in which higher [PdxK] increases the amount of PMP available to directly activate TyrB.

**FIGURE 7:**
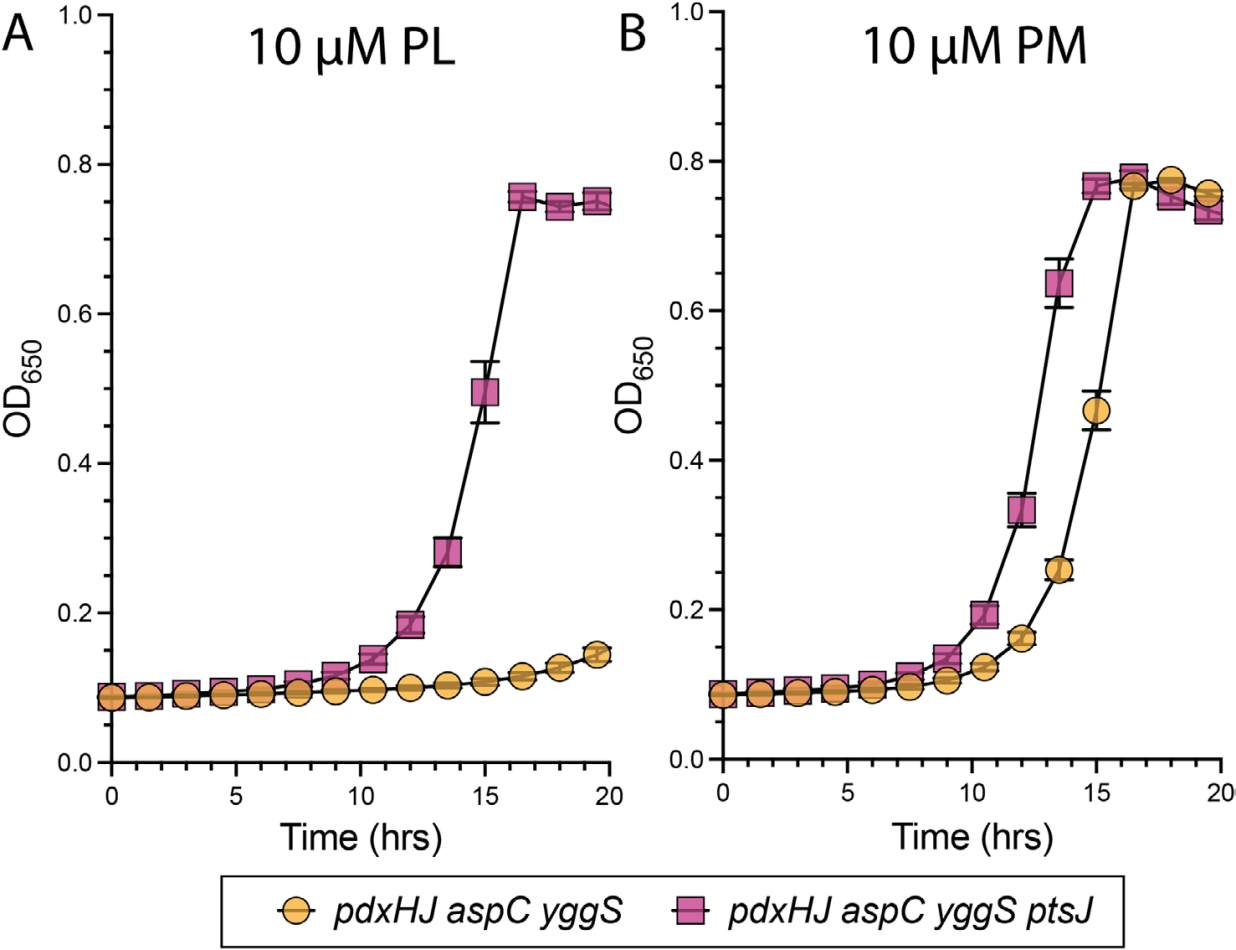
In the presence of pyridoxal, loss of PtsJ supports TyrB‐dependent aspartate synthesis in the absence of YggS. Growth of *pdxHJ aspC yggS* (circles, DM16705) and *pdxHJ aspC yggS ptsJ* (squares, DM18755) mutant strains on minimal media supplemented with (A) 10 μM PL or (B) 10 μM PM. Error bars represent the standard deviation of three biological replicates.

A *pdxJH aspC yggS ptsJ* mutant strain can grow with 1 or 10 μM PL, while the parental strain (*pdxJH aspC yggS*) fails to grow with up to 10 μM PL (Figure 7, Figure S5). Thus, a combination of exogenous PL and increased [PdxK] suppresses the effect of a *yggS* mutation in the *pdxJH aspC* background. It is possible that increased PdxK generates a larger intracellular pool of PLP that improves maturation of TyrB with free PLP. However, PdxK is feedback‐inhibited by the PLP it produces, so it seems unlikely that free PLP would appreciably accumulate. These results are also consistent with the working model in which PdxK●PLP can mature TyrB inefficiently (Figure 4). In this scenario, increasing the amount of PdxK●PLP activates TyrB by mass action, despite a more efficient route being dependent on YggS. This would suggest functional overlap between PdxK and YggS, a notion consistent with studies reporting the ability of both PdxK and YggS to traffic PLP to PLP‐DEs in vitro (Deka et al. 2019; Farkas and Fitzpatrick 2024; Ghatge et al. 2012; Kim et al. 1988; Tramonti et al. 2022). In light of abundant evidence of PLP transfer by PdxK and increasing evidence for PLP transfer by YggS, we favor the later scenario but cannot exclude the former.

**FIGURE 8:**
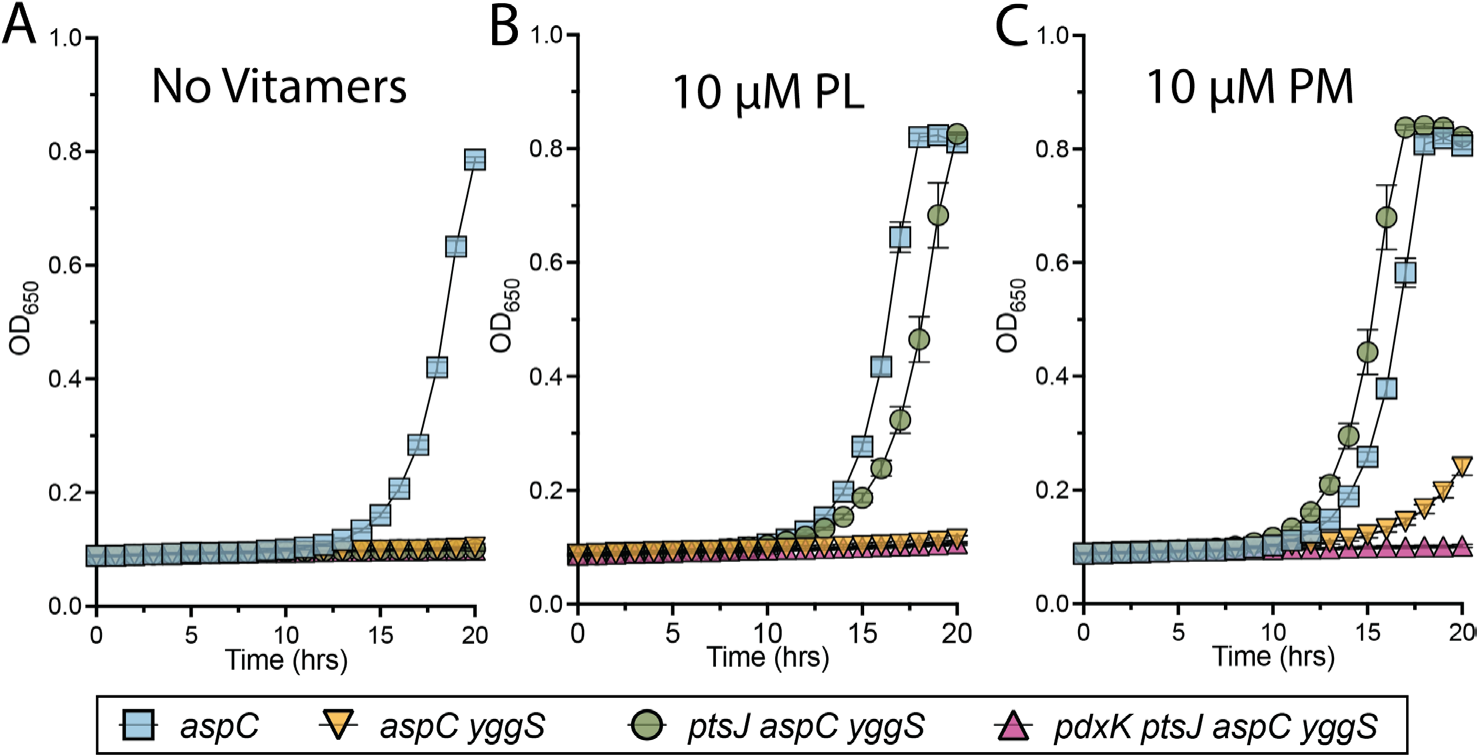
Upregulation of PdxK restores growth of *aspC yggS* mutant only if vitamers are supplemented. Growth of *aspC* (squares, DM16409), *aspC yggS* (downward triangle, DM16841), *ptsJ aspC yggS* (circles, DM18753), and *pdxK ptsJ aspC yggS* (upward triangle, DM18784) in minimal media with (A) no addition, (B) 10 μM PL or (C) 10 μM PM. Error bars represent the standard deviation of three biological replicates.

The impact of increased [PdxK] in strains with a functional *de novo* PLP synthetic pathway was assessed. In the absence of vitamers, a *ptsJ* mutation does not alleviate the growth defect of an *aspC yggS* mutant (Figure 8A). This result is explained by the kinase activity of PdxK; since endogenously synthesized vitamers are already phosphorylated, they are not substrates of PdxK. In contrast, when *de novo* synthesis is functional and PL or PM are provided, a *ptsJ* mutation restores growth of the *aspC yggS* mutant (Figure 8). This result confirms that if PdxK has suitable substrates, it can facilitate TyrB‐dependent aspartate synthesis in the absence of YggS. In the case of PM, we suggest increased PdxK‐derived PMP allows maturation of TyrB by the mechanism outlined above. We suggest that PL supplementation in the *ptsJ aspC yggS* background allows YggS‐independent TyrB maturation by increasing the amount of [PdxK•PLP] or [PLP] available to activate TyrB. This result shows that when *de novo* biosynthesis is functioning, PdxK is unable to facilitate sufficient generation of TyrB•PLP for TyrB‐dependent aspartate biosynthesis. The positive effect of a *ptsJ* mutation on growth when PL is provided indicates that increased [PdxK•PLP] or [PLP] can restore maturation of TyrB in the absence of YggS. As expected, the effect of the *ptsJ* mutation is dependent on *pdxK* (Figure 8).

### 2.7 Testing the Working Model for the Role of YggS in TyrB Maturation

In total, results here and in the literature are consistent with the working model depicted in Figure 4 and summarized above. The main principles of this model are that TyrB can be matured by protein‐mediated PLP carriers as well as free PLP or PMP, but crucially, these pathways are not equally efficient under all conditions.

**FIGURE 9:**
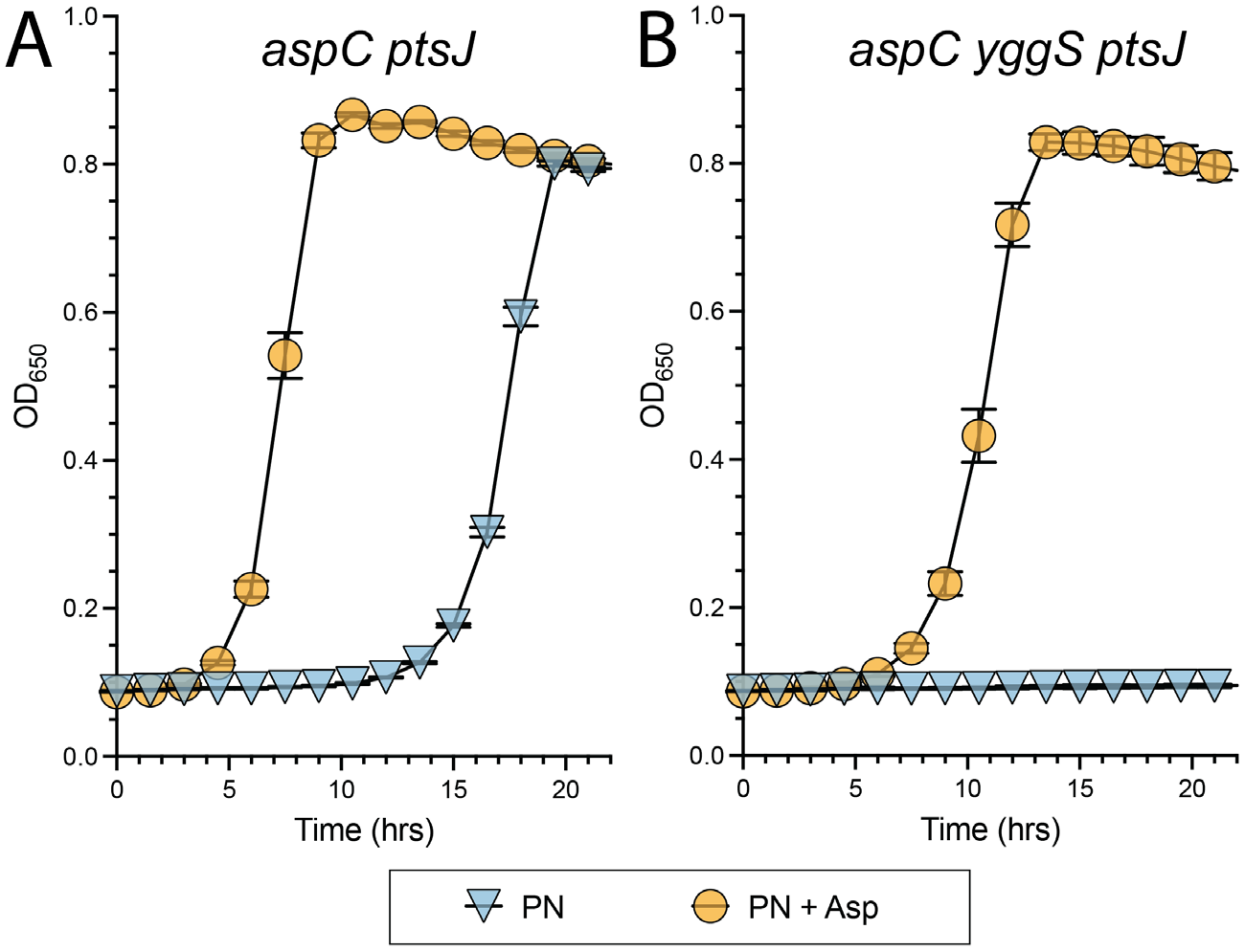
The aspartate requirement of an *aspC yggS ptsJ* strain is not rescued by pyridoxine. Growth of (A) *aspC ptsJ* (DM18752) or (B) *aspC yggS ptsJ* (DM18753) mutant strains in minimal glycerol media supplemented with 10 μM PN in the presence (circles) or absence (triangles) of 1.8 mM aspartate. Error bars represent the standard deviation of three biological replicates.

A prediction of this model is that upregulation of PdxK will not restore TyrB‐dependent growth to an *aspC yggS* mutant when PN is provided as the sole B_6_ vitamer. Phosphorylation of PN by PdxK generates PNP. Unlike PLP or PMP, PNP cannot be used to directly activate TyrB and must be oxidized to PLP by PdxH. Satisfyingly, 1 or 10 μM PN does not restore growth to an *aspC yggS ptsJ* mutant (Figure 9, data not shown for 1 μM PN). Thus, PLP derived from PdxH does not mature TyrB efficiently enough for sufficient aspartate production in the absence of YggS.

### 2.8 Conclusions

This study maps multiple in vivo pathways for maturation of the aminotransferase TyrB and supports a role for YggS as an intermediate PLP carrier. The data indicate that YggS specifically mediates PLP transfer from PdxH or ‐K to TyrB. The data complement and extend in vitro studies by providing strong evidence that YggS is involved in the in vivo maturation of PLP‐DEs. The exact mechanism of PLP transfer by YggS remains to be determined. It is possible that transfer is mediated by direct protein–protein interactions or that YggS is simply a protein with an intermediate affinity for PLP and serves as a protective intermediate in the cytosolic PLP binding equilibrium.

While in vitro experiments show that PdxH and PdxK can transfer PLP to PLP‐DEs, the role for this transfer in vivo remains unclear. In vivo results herein indicate that transfer of PLP from PdxH to TyrB occurs inefficiently. However, these results leave open the possibility that PdxK, when overexpressed, can efficiently transfer PLP to TyrB. Although YggS may act as an intermediate carrier between PLP‐synthesizing enzymes and TyrB, other PLP‐DEs may efficiently obtain PLP from PdxH and/or ‐K in vivo without a YggS intermediate. Further studies are needed to test this possibility. A redundancy of PLP carrier proteins would suggest that PLP‐DEs could have unique and/or condition‐dependent requirements for specific carriers, analogous to what has been proposed for FeS carrier proteins and their clients (Esquilin‐Lebron et al. 2021).

Although this study uncovered a role for PLPHP homolog YggS in the maturation of TyrB, this relatively mundane PLP‐DE cannot be the sole client of this highly conserved protein. Based on the pleiotropic effect of PLPHP mutations on PLP‐dependent metabolism (Ciapaite et al. 2023; Darin et al. 2016; Farkas and Fitzpatrick 2024; Ito et al. 2019; Johnstone et al. 2019; Vu et al. 2020), PLPHP homologs are likely to facilitate maturation of numerous PLP‐DEs. Thus, an extension of our working model suggests that there will be lower cofactor occupancy in many PLP‐DEs in a *yggS* mutant. Largely due to the difficulty of recapitulating in vivo conditions in in vitro experiments, this prediction has not yet been satisfactorily tested.

The model proposed herein explains why deleting *yggS* fails to produce a dramatic defect in most strain backgrounds. A bacterial cell is expected to tolerate inefficient trafficking and loading of PLP when the normal cadre of enzymes is available. This expectation aligns with knowledge that the bacterial cell is generally overprogrammed (Sander et al. 2019), and that numerous enzymes provide function sufficient for growth with significantly < 100% activity (Christopherson et al. 2012; Downs et al. 2018; Fendt et al. 2010; Hackett et al. 2016; Koenigsknecht et al. 2012; Schmitz and Downs 2004). Thus, metabolic robustness complicates efforts to identify the clients of PLPHP and other putative PLP carriers. This study exploited a condition where lack of efficient enzyme maturation leads to a growth defect. In an *aspC* mutant, growth requires aspartate production by TyrB, which is a low abundance protein with poor AspAT activity. Since cells maintain a larger pool of aspartate than of all aromatic amino acids combined (Bennett et al. 2009), efficient cofactor loading is required for TyrB to provide sufficient aspartate for growth. While TyrB is a poor AspAT, it is a proficient aromatic amino acid aminotransferase (AAT). Thus, the cell can tolerate inefficiency in cofactor loading when TyrB is required only for AAT activity, but not when it acts as the sole AspAT.

Genetic analyses suggest that YggS is required for the efficient maturation of TyrB, yet in vitro TyrB activity was no different in crude extracts carrying or lacking YggS. Additionally, while transfer of PLP from YggS to TyrB was shown with purified proteins, the maturation of TyrB via YggS●PLP was less efficient than free PLP in vitro. The apparent discrepancy between the in vivo and in vitro results may reflect practical differences in the availability of PLP in the cytosol vs. buffer‐based assay environments. Neither the crude extract assay nor the purified protein assay accurately reproduce in vivo conditions (Kontopidis and Patergiannakis 2025). Preparation of crude extracts necessarily dilutes cellular contents and otherwise alters the molecular milieu. In the dilute assay buffer or crude extract, free PLP matures apo‐enzymes via diffusion. However, in a crowded, metabolically‐active cytosol, apo‐enzymes may struggle to efficiently retrieve free PLP due to its propensity to form Schiff bases with off‐target components and/or activity of cellular PLP phosphatases. Such “off target” interactions and dephosphorylation events would decrease diffusion‐based maturation of apo‐enzymes. While diffusion‐based maturation is expected to occur at some rate in vivo, we suggest that carrier‐mediated maturation is more efficient. When the metabolic network is perturbed, such as in the *aspC* mutant described here, inefficient maturation can result in compromised growth.

A study addressing the ability of putative PLP carrier PdxH to transfer PLP to apo‐serine hydroxymethyltransferase (GlyA) suggests a strategy moving forward (Yang and Schirch 2000). These authors showed that while PLP matured apo‐GlyA in buffer, addition of lyophilized cell extract decreased the efficiency of maturation. In this simulated in vivo condition, PdxH‐mediated PLP transfer outperformed maturation with free PLP. Similar experiments with YggS could provide insights into the mechanism and kinetics of PLP transfer by YggS.

Past studies and the study herein show that the metabolic network must be perturbed to detect a physiological requirement for the PLPHP homolog, YggS. As such, we consider PLPHP to be in a class of proteins that can be described as metabolic modulators. In this context, metabolic modulators are proteins that optimize metabolic network function, but do not always impact growth in the laboratory setting. These proteins are presumed to be crucial for broad competitive fitness, but cellular overprogramming, redundancy, and robustness can obscure the role they have in the laboratory setting.

## 3 Materials and Methods

### 3.1 Strains, Media and Chemicals

Strains used in this study are derivatives of *Salmonella enterica* serovar Typhimurium LT2 (denoted *S. enterica* throughout) and are listed in Table S3. When required, strains were constructed by P22 (*HT105/1 int‐201*) transduction, using standard protocols (Downs and Petersen 1994; Schmieger 1972). *S. enterica* strains were routinely grown in nutrient broth (NB; Difco NB mix; 8 g/L, 5 g/L NaCl) supplemented with 1 μM PL as rich medium. Minimal medium was No Carbon Energy (NCE) media supplemented with MgSO4 (1 mM), trace elements (1×) and glycerol (22 mM) as the sole carbon source (Balch and Wolfe 1976; Davis et al. 1980; Vogel and Bonner 1956). *E. coli* was cultured in super broth (SB, 2 g/L Bacto tryptone, 20 g/L yeast extract, 5 g/L NaCl, 5 mM NaOH) supplemented with kanamycin (50 μg/mL). Other supplements were added as needed in the concentrations noted in the text and/or figure legends. All chemicals were purchased from MilliporeSigma (St. Louis, MO) unless otherwise stated.

### 3.2 Growth Analyses

Single colonies were used to inoculate 1 mL NB with 1 μM PL in biological triplicate. Cultures were incubated overnight at 37°C with shaking (250 rpm) in an Innova 43 incubator. Cells were pelleted at 3200 rpm for 15 min at room temperature in a Model GCC‐S clinical centrifuge (Globe Scientific, Mahwah, NJ). Each cell pellet was resuspended in 1 mL 0.85% saline. An aliquot (4 μL) of the saline cell suspensions was used to inoculate 196 μL of minimal media (2% (vol/vol)) in a 96 well plates (Corning). Plates were sealed with Parafilm and incubated at 37°C with shaking (medium setting) in a BioTek ELx808 plate reader (BioTek Instruments, Winooski, VT). Cell growth was measured as the absorbance at 650 nm. Data were visualized using Prism 10 (GraphPad Software, La Jolla, CA).

### 3.3 Isolation of Null Allele of *pdxK*

Strains lacking *ptsJ* are sensitive to the PN analog, 4‐deoxypyridoxine (dPN). The sensitivity is easily suppressed by loss of PdxK function (Vu and Downs 2022). This phenotype was used to isolate a null allele of *pdxK* in an *aspC yggS ptsJ* background. DM18753 was plated on NCE glucose minimal media containing 10 μM dPN and 1.8 mM aspartate. Spontaneous dPN‐resistant suppressor mutants were isolated, phenotypically confirmed, and their *pdxK* locus sequenced (Eurofins Genomics, Louisville, KY). Of all three suppressors sequenced, each contained a unique mutation in *pdxK*. The *pdxK* allele in suppressor mutant DM18784 (*pdxK*694) had a 14 bp insertion after nucleotide 464 which introduced a frameshift and encoded a truncated PdxK protein.

### 3.4 RNA Extraction

Single colonies of DM16409 and DM16841 were used to inoculate 2 mL NB with 1 μM PL in biological triplicate. Cultures were incubated at 37°C with shaking for 8 h. Cells were then pelleted and suspended in 2 mL saline (0.85%). Saline‐resuspended cells were subcultured (1:100) in 3 mL of minimal glycerol media supplemented with aspartate (1.8 mM). Cultures were incubated at 37°C with shaking for 12 h before pelleting, resuspension in 3 mL saline, and subculturing (1:100) into 10 mL minimal glycerol media containing aspartate (1.8 mM). These cultures were incubated at 37°C with shaking until they reached OD_650_ ~0.6–0.7. Cells were harvested by centrifugation at 17,000xg for 5 min (4°C) and the pellets were flash frozen in liquid nitrogen and stored at −80°C until use.

RNA was extracted using the RNASnap method described previously (Stead et al. 2012). Cell pellets were resuspended in 150 μL boil solution [EDTA (0.5 M, pH 8), SDS (0.025%, mass/vol), β‐mercaptoethanol (1%), formamide (95%, vol/vol)] and lysed by incubation at 95°C for 7 min. Lysates were cleared by centrifugation at 17,000×*g* for 5 min (22°C); 100 μL of the supernatant was transferred to a new tube containing 400 μL of H_2_O and 50 μL sodium acetate solution (3 M). Nucleic acids were precipitated by adding 1600 μL of 100% ethanol to each sample before incubating at −80°C for 1 h. Nucleic acids were pelleted by centrifugation at 16,000×*g* for 30 min (4°C). Pellets were washed with 300 μL 70% ethanol before pelleting at 8000×*g* for 8 min (4°C). Supernatants were removed, pellets were air dried and resuspended in 100 μL of H_2_O. Water‐insoluble components were removed by pelleting at 16,000×*g* for 1 min, and 90 μL of the supernatant was transferred to a new tube. Nucleic acid samples were treated with DNase using the TURBO DNase DNA‐free kit per manufacturer's instructions (Invitrogen, Waltham, MA).

RNA was purified by ethanol precipitation, as described above. RNA was quantified using the Qubit RNA Broad Range kit (Invitrogen). All RNA samples possessed an RNA integrity and quality (IQ) score ≥ 8.7 as determined by the Qubit RNA IQ assay kit (Invitrogen). RNA samples (1 μg) were promptly reverse transcribed into cDNA using the iScript cDNA synthesis kit per manufacturer's instructions (BioRad, Hercules, CA). cDNA was stored at −20°C until use.

### 3.5 Real Time Quantitative PCR

RT‐qPCR was performed using a 96 well optical plate and a 7500 Fast Real Time PCR system (Applied Biosystems, Waltham, MA). Each 20 μL RT‐qPCR reaction contained 1× Fast SYBR green master mix (Applied Biosystems, Waltham, MA), 0.5 μM of each gene specific primer (Table S4), and 15 ng of cDNA. Melting curve analysis ensured that each reaction generated only one PCR product. For each gene, the average cycle threshold (Ct) of each biological replicate was calculated by taking the mean of three technical replicates. Expression of *tyrB* was normalized to reference gene, *gyrA*, by calculating ΔCt for each of three biological replicates. To determine the fold change in *tyrB* expression between strains, the comparative threshold cycle (ΔΔCt) method was utilized (Livak and Schmittgen 2001). The resulting 2^−ΔΔCt^ values for each strain were compared using a two‐tailed Welch's *t*‐test in Prism 10 (GraphPad).

### 3.6 Purification of YggS and TyrB

SeTyrB protein with a C‐terminal 5xHis tag was previously purified (Vu and Downs 2021b). YggS of *S. enterica* was expressed with a C‐terminal 5xHis tag from a modified pET28b(+) vector (Galloway et al. 2013). *E. coli* BL21AI carrying pET28b‐YggS was inoculated into 10 mL SB media containing kanamycin. Cultures were incubated at 37°C with shaking for 16 h before subculturing (1:300) into two baffled flasks containing 1.5 L SB with kanamycin. Cultures were incubated at 37°C with shaking in an Innova44 incubator (New Brunswick Scientific). At OD_650_ ~0.6, expression of YggS was induced with 0.2 mM IPTG and 0.2% arabinose. Induced cultures were incubated at 22°C for 16 h before harvesting at 5000×*g* for 20 m. Cell pellets were stored at −80°C until use.

Cell pellets were resuspended 2.5 mL per g in bind buffer (20 mM potassium phosphate buffer, 150 mM NaCl, 5 mM imidazole, pH 7.3) containing lysozyme (1 mg/mL) and DNase (0.125 mg/mL). Cells were lysed at 18,000 psi using a OneShot Cell Disruptor (Constant Systems, Daventry, UK). Phenylmethylsulfonyl fluoride (1 mM) was added to lysate. Lysate was cleared by centrifugation at 45,000×*g* for 45 m. Cleared lysates were filtered using a 0.45 μm polyvinylidene difluoride filter before application to an equilibrated 5 mL HisPur Ni‐NTA Superflow agarose column (Thermo Fisher Scientific, Waltham, MA). The column was washed with 5 column volumes (CV) of bind buffer and 5 CV of wash buffer (20 mM potassium phosphate buffer, 150 mM NaCl, 20 mM imidazole, pH 7.3). YggS was eluted using 5 CV of elution buffer (20 mM potassium phosphate buffer, 150 mM NaCl, 500 mM imidazole, pH 7.3). Upon SDS‐PAGE analysis, fractions containing the protein of interest were combined. Using an Amicon Ultra—15 centrifugal filter unit with a 10 kD molecular cutoff (MilliporeSigma), protein was concentrated and buffer exchanged into buffer A (50 mM HEPES, 50 mM NaCl, 10% glycerol, pH 7.5). Protein was desalted using a PD‐10 column (Cytiva, Marlborough, MA) and eluted using 3.5 mL of buffer A. Protein concentration was determined using a Pierce Bicinchoninic Acid (BCA) Protein Assay kit (Thermo Fisher Scientific). Protein purity was determined to be > 95% via SDS‐PAGE analysis (Figure S6). Protein was flash‐frozen and stored at −80°C until use.

### 3.7 Preparation of Apo‐TyrB and Apo‐YggS

Apo proteins were prepared by diluting protein to 200 μM in freshly prepared buffer B (50 mM HEPES, 50 mM NaCl, 100 or 300 mM L‐cysteine HCl, pH 7.5). Proteins were incubated with cysteine for 1 h. Using an Amicon Ultra—15 centrifugal filter unit with a 10 kD molecular cutoff (MilliporeSigma), proteins were concentrated and buffer exchanged three times using 10 mL of buffer A. Concentrated protein was applied to a PD‐10 column (Cytiva, Marlborough, MA) and eluted with 3.5 mL of buffer A. The concentration of TyrB was determined via BCA protein assay. Concentrations of YggS was determined using a NanoDrop Spectrophotometer (*ε*_280_ = 18450 M^−1^ cm^−1^) (Gasteiger et al. 2003).

Upon addition of 100 mM cysteine, TyrB underwent a visible color change (yellow to clear)–supporting the elimination of bound PLP (Tramonti et al. 2022). The successful generation of apo‐TyrB was confirmed by its low AspAT activity. The yellow color of YggS did not change after addition of up to 300 mM cysteine. A decrease in protein‐bound PLP can be visualized as a decline in absorbance at 422 nm (Tramonti et al. 2022); the UV spectra of YggS was not significantly changed after cysteine treatment (Figure S6). To estimate the apo/holo ratio, 150 μM of YggS was denatured in 0.1 M NaOH to release PLP. Using a SpectraMax Abs Plus spectrophotometer (Molecular Devices, San Jose, CA) and a quartz microwell plate, the concentration of PLP was determined using A_388_ (normalized to 1 cm) and the extinction coefficient of PLP in 0.1 M NaOH (*ε*_388_ = 6600 M^−1^ cm^−1^) (Peterson and Sober 1954).

### 3.8 PLP Transfer Assay

AspAT activity of the apo‐TyrB preparation was determined via coupled enzyme assay (Arnold and Parslow 1995; Vu and Downs 2021b) after incubation with PLP or YggS protein. Reactions (200 μL) contained apo‐TyrB (4 μM, monomeric concentration), porcine malate dehydrogenase (0.025 U/μL), NADH (0.4 mM), α‐ketoglutarate (5 mM) in HEPES buffer (50 mM, pH 7.8). Upon addition of YggS (40 μM, monomeric concentration) or free PLP (40 μM), reactions were mixed and incubated at room temperature for 10 m. AspAT reactions were initiated with addition of aspartate (100 mM) or buffer (for control reactions) in a quartz microwell plate. Reactions were monitored at room temperature using a SpectraMax Abs Plus spectrophotometer. Change in A_340_ per min was determined within the linear range (30–60 s for TyrB with free PLP, 120–150 s for all other reactions), and converted to rate of NADH oxidation using the extinction coefficient of NADH (*ε*_340_ = 6220 M^−1^ cm^−1^) (Vu and Downs 2021b). Each reaction was performed in technical triplicate. Statistical analysis was performed using Prism 10 (GraphPad).

## Author Contributions

**Brandi A. Buckner:** conceptualization, methodology, data curation, investigation, writing – original draft, writing – review and editing. **Diana M. Downs:** conceptualization, supervision, funding acquisition, project administration, writing – review and editing.

## Funding

This work was supported by the National Institutes of Health, R35GM153189, 1T32GM142623.

## Disclosure

The authors have nothing to report.

## Ethics Statement

The authors have nothing to report.

## Conflicts of Interest

The authors declare no conflicts of interest.

## Supporting information


**Figure S1:** Eliminating PNP production via *pdxB* does not eliminate the growth defect observed in *aspC yggS* strains. Growth of *pdxB aspC* (circles, DM18712) and *pdxB aspC yggS* (squares, DM18713) strains in minimal media supplemented with 10 μM PL. Error bars represent the standard deviation of three biological replicates.
**Figure S2:** Supplementation of glutamate or oxaloacetate does not rescue *aspC yggS* strain. Growth of *aspC* (circles, DM16409) and *aspC yggS* (triangles, DM16841) in (A) minimal media containing (B) 1.8 mM glutamate (glu), (C) 1.8 mM oxaloacetate (OAA) or (D) both. Error bars represent the standard deviation of three biological replicates.
**Figure S3:** The status of YggS does not affect the B_6_ vitamer requirement of a B_6_ auxotroph. Growth of *pdxHJ* (squares, DM16397) and *pdxHJ yggS* (circles, DM16557) strains in minimal glycerol media supplemented with (A) 1 μM PL or (B) 1 μM PM. Error bars represent the standard deviation of three biological replicates.
**Figure S4:**. Upregulation of *tyrB* rescues growth of *pdxH aspC yggS* strain. Growth of *pdxH aspC yggS* (circles, DM16840) and *pdxH aspC yggS tyrR* (squares, DM18569) in minimal glycerol media supplemented with (A) 1 μM PL or (B) 1 μM PM. Error bars represent the standard deviation of three biological replicates.
**Figure S5:** Upregulation of PdxK allows growth of *pdxHJ aspC yggS* on low vitamer concentrations. Growth of *pdxHJ aspC yggS* (circles, DM16705) and *pdxHJ aspC yggS ptsJ* (squares, DM18755) mutant strains on minimal media supplemented with (A) 1 μM PL or (B) 1 μM PM. Error bars represent the standard deviation of three biological replicates.
**Figure S6:** Preparation of YggS protein. (A) Purified YggS protein visualized by Coomaisee blue staining of a 10% SDS‐PAGE gel. (B) UV spectra of untreated and cysteine‐treated YggS (150 μM) in 50 mM Tris buffer (pH 7.6). Error bars represent standard deviation of three technical replicates. (C) UV spectra obtained after YggS protein (150 μM) was denatured in 0.1 M NaOH to release protein‐bound PLP. Error bars represent standard deviation of three technical replicates.
**Table S1:** Expression of *tyrB* is not affected by YggS status. RT‐qPCR was used to compare expression of *tyrB* in *aspC (*DM16409) and *aspC yggS (*DM16841) strains during growth in minimal media supplemented with aspartate. Expression of *tyrB* was normalized to reference gene *gyrA*. Average threshold (Ct) and relative fold change were calculated using three biological replicates as described in the Material and Methods.
**Table S2:** Summary and interpretation of key results from growth analyses. Observations critical to the construction of the working model are simply presented with the interpretations they allowed. The figure with the relevant data is indicated in each case, as is the vitamer involved. This table does not present all the relevant data that went into defining the model, which is described and shown in the manuscript text.
**Table S3:** Strains and plasmids.
**Table S4:** Primers used for RT‐qPCR.

## Data Availability

All relevant data are included in the content of this manuscript.
