# Supplementary material for: PLP Homeostasis Protein Is Required for Efficient Maturation of Aromatic Amino Acid Aminotransferase in Salmonella enterica
Source: Mol Microbiol. 2026 Apr 27;126(1):1–14. doi: 10.1111/mmi.70072 (PMC13353753; doi:10.1111/mmi.70072)
Supplement: Supplementary file 1 — Figure S1: Eliminating PNP production via pdxB does not eliminate the growth defect observed in aspC yggS strains. Growth of pdxB aspC (circles, DM18712) and pdxB aspC yggS (squares, DM18713) strains in minimal media supplemented with 10 μM PL. Error bars represent the standard deviation of three biological replicates. Figure S2: Supplementation of glutamate or oxaloacetate does not rescue aspC yggS strain. Growth of aspC (circles, DM16409) and aspC yggS (triangles, DM16841) in (A) minimal media containing (B) 1.8 mM glutamate (glu), (C) 1.8 mM oxaloacetate (OAA) or (D) both. Error bars represent the standard deviation of three biological replicates. Figure S3: The status of YggS does not affect the B6 vitamer requirement of a B6 auxotroph. Growth of pdxHJ (squares, DM16397) and pdxHJ yggS (circles, DM16557) strains in minimal glycerol media supplemented with (A) 1 μM PL or (B) 1 μM PM. Error bars represent the standard deviation of three biological replicates. Figure S4:. Upregulation of tyrB rescues growth of pdxH aspC yggS strain. Growth of pdxH aspC yggS (circles, DM16840) and pdxH aspC yggS tyrR (squares, DM18569) in minimal glycerol media supplemented with (A) 1 μM PL or (B) 1 μM PM. Error bars represent the standard deviation of three biological replicates. Figure S5: Upregulation of PdxK allows growth of pdxHJ aspC yggS on low vitamer concentrations. Growth of pdxHJ aspC yggS (circles, DM16705) and pdxHJ aspC yggS ptsJ (squares, DM18755) mutant strains on minimal media supplemented with (A) 1 μM PL or (B) 1 μM PM. Error bars represent the standard deviation of three biological replicates. Figure S6: Preparation of YggS protein. (A) Purified YggS protein visualized by Coomaisee blue staining of a 10% SDS‐PAGE gel. (B) UV spectra of untreated and cysteine‐treated YggS (150 μM) in 50 mM Tris buffer (pH 7.6). Error bars represent standard deviation of three technical replicates. (C) UV spectra obtained after YggS protein (150 μM) was denatured in 0.1 M NaOH to r [file MMI-126-1-s001.docx]

**Supplemental Materials**

PLP Homeostasis Protein is required for efficient maturation of aromatic amino acid aminotransferase in *Salmonella enterica*.

Brandi A. Buckner and Diana M. Downs*

Department of Microbiology

University of Georgia

Athens, GA, USA

*Corresponding Author: Diana M. Downs, dmdowns@uga.edu

Keywords: Vitamin B6, pyridoxamine, transamination, pyridoxal 5’-phosphate (PLP), PLP Homeostasis Protein (PLPHP), YggS

| Normalized Expression of *tyrB* | | |
| --- | --- | --- |
| Strain | Relative change in expression  (2^-ΔΔCt^  ± standard deviation) | p-value  (Welch’s T-test) |
| *aspC* | 1.01 ± 0.13 | 0.0921 |
| *aspC yggS* | 0.69 ± 0.20 |  |

**Table S1. Expression of *tyrB* is not affected by YggS status.** RT-qPCR was used to compare expression of *tyrB* in *aspC (*DM16409) and *aspC yggS (*DM16841) strains during growth in minimal media supplemented with aspartate. Expression of *tyrB* was normalized to reference gene *gyrA*. Average threshold (Ct) and relative fold change were calculated using three biological replicates as described in the Material and Methods.

| Observation | Interpretation | Source | Vitamer |
| --- | --- | --- | --- |
| A *pdxHJ* mutant requires 1 μM PM, while a *pdxHJ aspC* mutant requires 10 μM PM, or 1 μM PM + aspartate | Synthesis of sufficient aspartate by TyrB requires high PM, while low PM satisfies the rest of the cellular B_6_ requirement. | Figure 3 | PM |
| A *pdxHJ aspC* *yggS* mutant requires 10 μM PM, or 1 μM PM + aspartate. | YggS is not required for maturation of TyrB with PM. | Figure 6 | PM |
| A *pdxHJ aspC* mutant requires 1 μM PL. | Low PL supports synthesis of sufficient aspartate by TyrB in the presence of YggS. | Figure 3 | PL |
| A *pdxHJ aspC* *yggS* mutant requires aspartate. Without aspartate, even 10 μM PL fails to allow growth. | Efficient maturation of TyrB with PLP requires YggS. | Figure 5 | PL |
| Increased PdxK allows YggS-independent maturation of TyrB with PL. | PdxK increases the PLP available to mature TyrB, independent of YggS.  or  Increased PdxK•PLP delivers PLP to TyrB independent of YggS. | Figure 7 | PL |

**Table S2. Summary and interpretation of key results from growth analyses.** Observations critical to the construction of the working model are simply presented with the interpretations they allowed. The figure with the relevant data is indicated in each case, as is the vitamer involved. This table does not present all the relevant data that went into defining the model, which is described and shown in the manuscript text.

**Table S3.** Strains and Plasmids.

| **Strain** | **Genotype** | **Source** |
| --- | --- | --- |
| DM16409 | *aspC832*::Tn10d(Tc)^1^ | Downs Laboratory |
| DM16841 | *yggS648*::Km *aspC832*::Tn10d(Tc) | Downs Laboratory |
| DM16839 | Δ*pdxH677* *aspC832*::Tn10d(Tc) | Downs Laboratory |
| DM16840 | Δ*pdxH677* *aspC832*::Tn10d(Tc) *yggS648*::Km | Downs Laboratory |
| DM16702 | Δ*pdxJ664* *aspC832*::Tn10d(Tc) | Downs Laboratory |
| DM16703 | Δ*pdxJ664* aspC*832*::Tn10d(Tc) *yggS648*::Km | Downs Laboratory |
| DM16704 | Δ*pdxJ664* Δ*pdxH673* *aspC832*::Tn10d(Tc) | Downs Laboratory |
| DM16705 | Δ*pdxJ664* Δ*pdxH673* *aspC832*::Tn10d(Tc) *yggS648*::Km | Downs Laboratory |
| DM15769 | *pdxB663*::Cm | Downs Laboratory |
| DM18712 | *aspC832*::Tn10(Tc) *pdxB663*::cm | This study |
| DM18713 | *aspC832*::Tn10(Tc) *pdxB663*::cm *yggS*648::km | This study |
| DM16397 | Δ*pdxJ664* Δ*pdxH673* | Downs Laboratory |
| DM16557 | ∆*pdxJ664* ∆*pdxH673* *yggS648*::Km | Downs Laboratory |
| DM16611 | *tyrR946*::Tn10d(Cm) | Downs Laboratory |
| DM18569 | Δ*pdxH677* *aspC832*::Tn10d(Tc) *yggS648*::Km *tyrR946*::Tn10(d)Cm | This study |
| DM16109 | *ptsJ*615::Cm | Downs Laboratory |
| DM18754 | Δ*pdxJ664* Δ*pdxH*673 *aspC832*::Tn10d(Tc) *ptsJ615*::Cm | This study |
| DM18755 | Δ*pdxJ664* Δ*pdxH673* *aspC832*::Tn10d(Tc) yggS*648*::Km *ptsJ615*::Cm | This study |
| DM18752 | *aspC832*::Tn10d(Tc) *ptsJ615*::Cm | This study |
| DM18753 | *aspC832*::Tn10d(Tc) *yggS648*::Km *ptsJ615*::Cm | This study |
| DM18784 | *aspC832*::Tn10d(Tc) *yggS648*::Km *ptsJ615*::Cm *pdxK*694 | This study |
| **Plasmid** | **Description** | **Reference** |
| pDM1574 | Modified pET28b(+) vector encoding SeYggS with C-terminal 5xHis Tag | (Galloway et al., 2013) |

^1^Tn*10*d refers to the transposition-defective mini-Tn*10* (Tn*10*Δ16Δ17) and encodes resistance to either tetracycline (Tc) or chloramphenicol (Cm) (Way et al., 1984).

Unless otherwise indicated, all gene deletion or gene insertions conferring chloramphenicol (Cm) or kanamycin (Km) resistance were generated using the Wanner method (Datsenko and Wanner, 2000).

**Table S4.** Primers used for RT-qPCR.

|  | Primer Name | Sequence (5’ to 3’) |
| --- | --- | --- |
| RT-qPCR of *gyrA* | gyrA_qPCR_F | GGGCGTTATCCTTATCCGCAC |
|  | gyrA.qPCR_R | GCCGTCGATAGCGTCGAGTTC |
| RT-qPCR of *tyrB* | TyrB_qPCR_F | TGCGCCGAATTTACTCCAGT |
|  | TyrB_qPCR_R | TTTAACGCCTCATCGCCCAG |

**
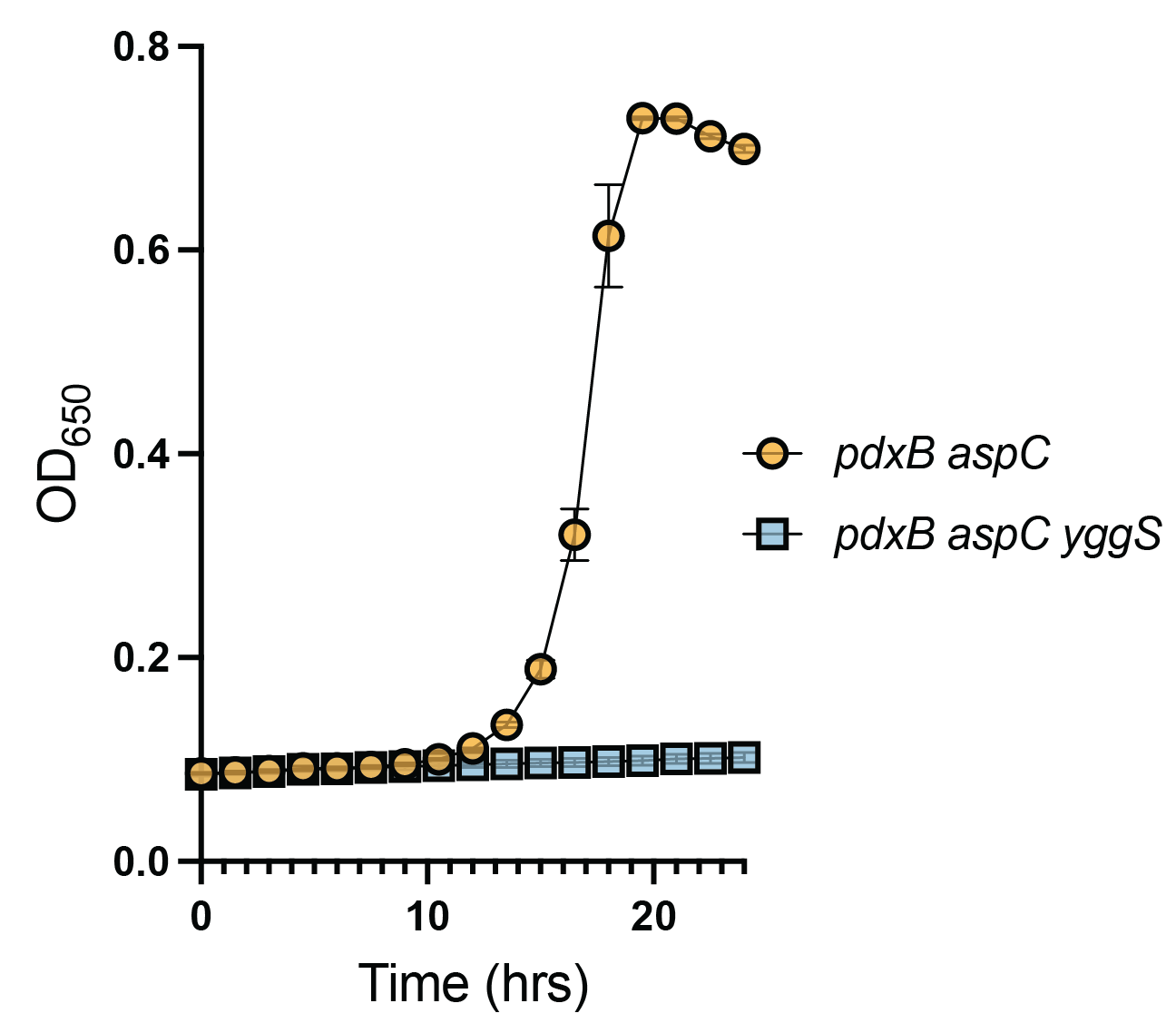
**

**Figure S1.** **Eliminating PNP production via *pdxB* does not eliminate the growth defect observed in *aspC yggS* strains.** Growth of *pdxB aspC* (circles, DM18712) and *pdxB aspC yggS* (squares, DM18713) strains in minimal media supplemented with 10 μM PL. Error bars represent the standard deviation of three biological replicates.

**
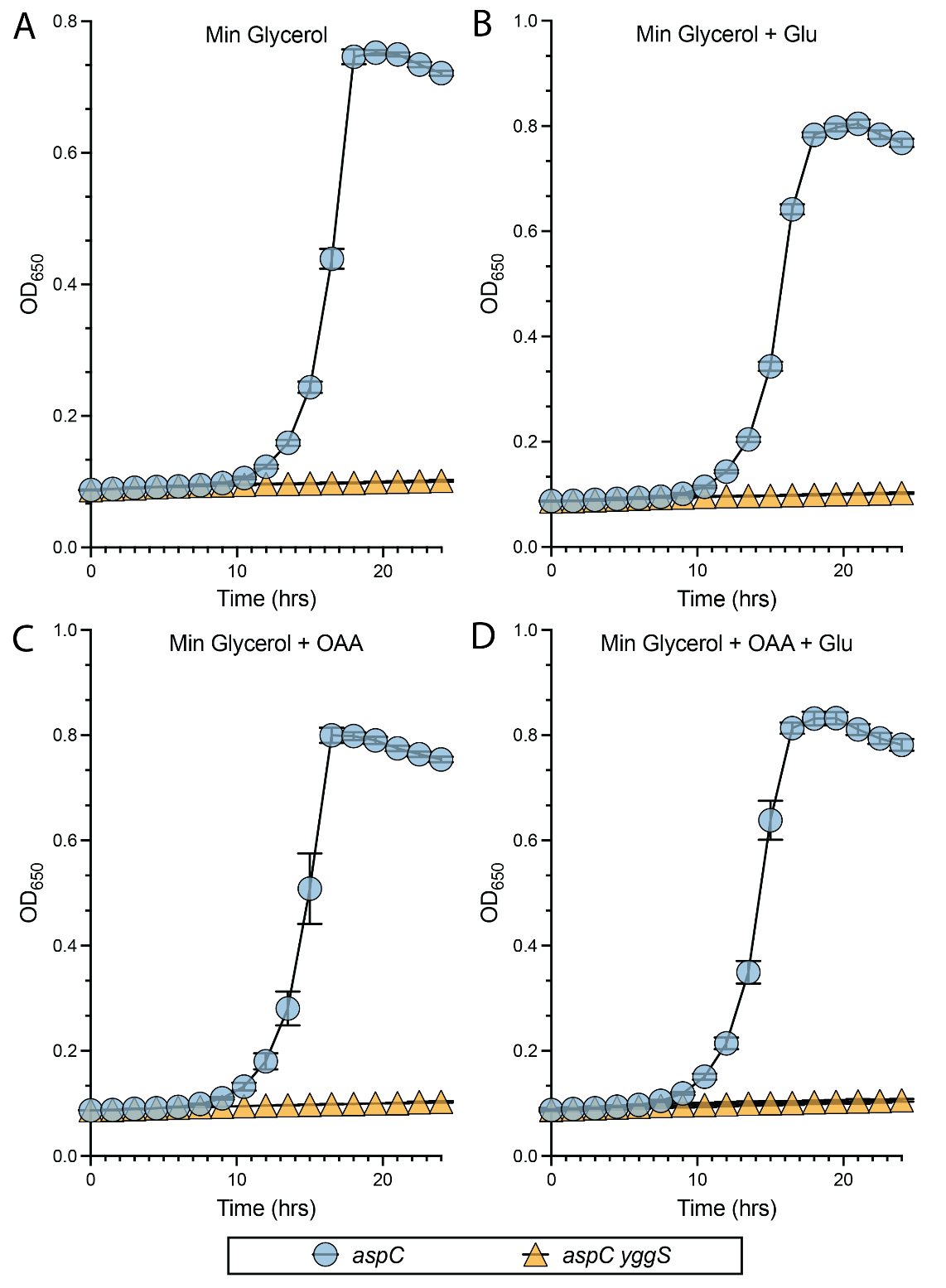
**

**Figure S2.** **Supplementation of glutamate or oxaloacetate does not rescue *aspC yggS* strain.** Growth of *aspC* (circles, DM16409) and *aspC yggS* (triangles, DM16841) in (A) minimal media containing (B) 1.8 mM glutamate (glu), (C) 1.8 mM oxaloacetate (OAA), or (D) both. Error bars represent the standard deviation of three biological replicates.

**
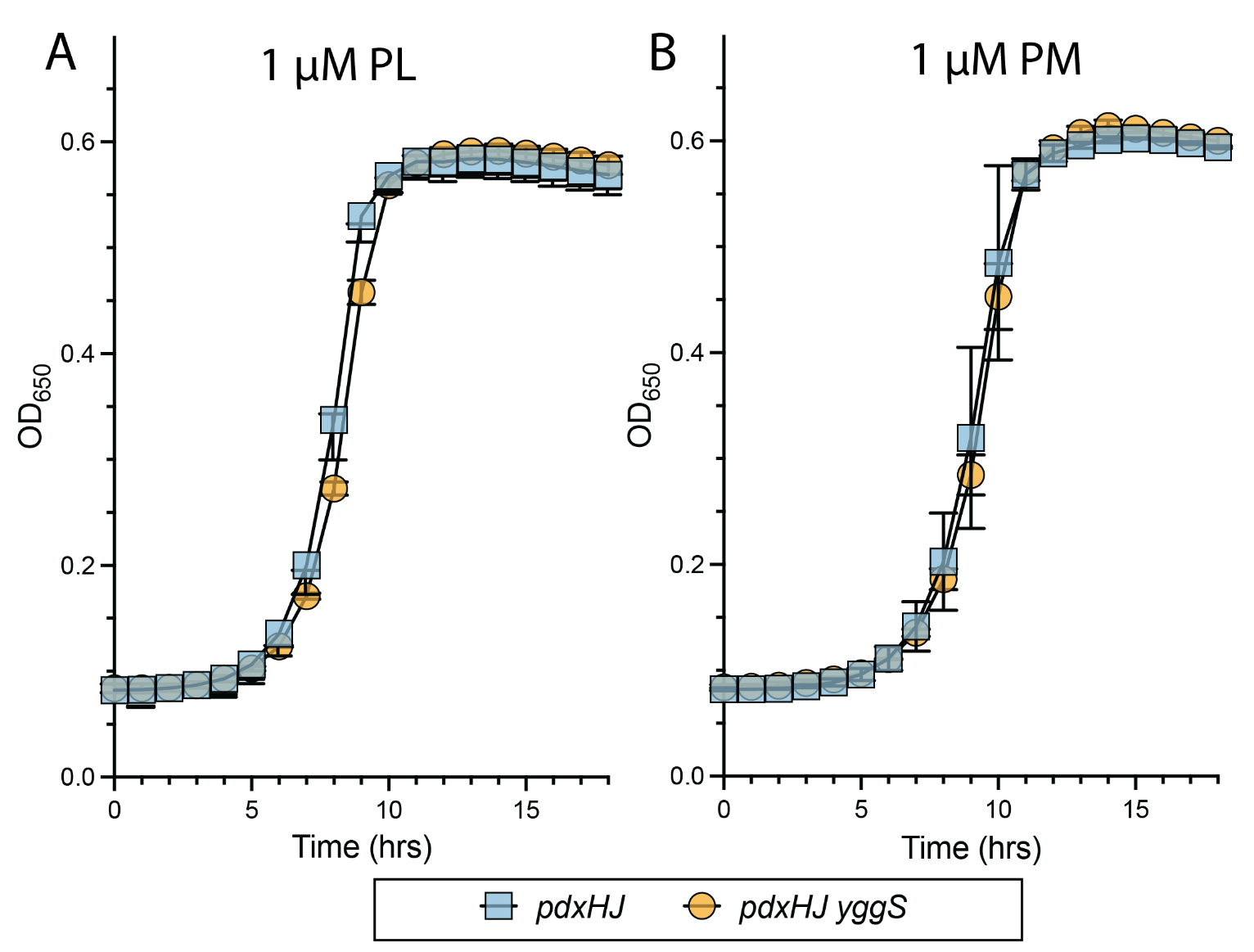
**

**Figure S3. The status of YggS does not affect the B_6_ vitamer requirement of a B_6_ auxotroph**. Growth of *pdxHJ* (squares, DM16397) and *pdxHJ yggS* (circles, DM16557) strains in minimal glycerol media supplemented with (A) 1 µM PL or (B) 1 µM PM. Error bars represent the standard deviation of three biological replicates.

**
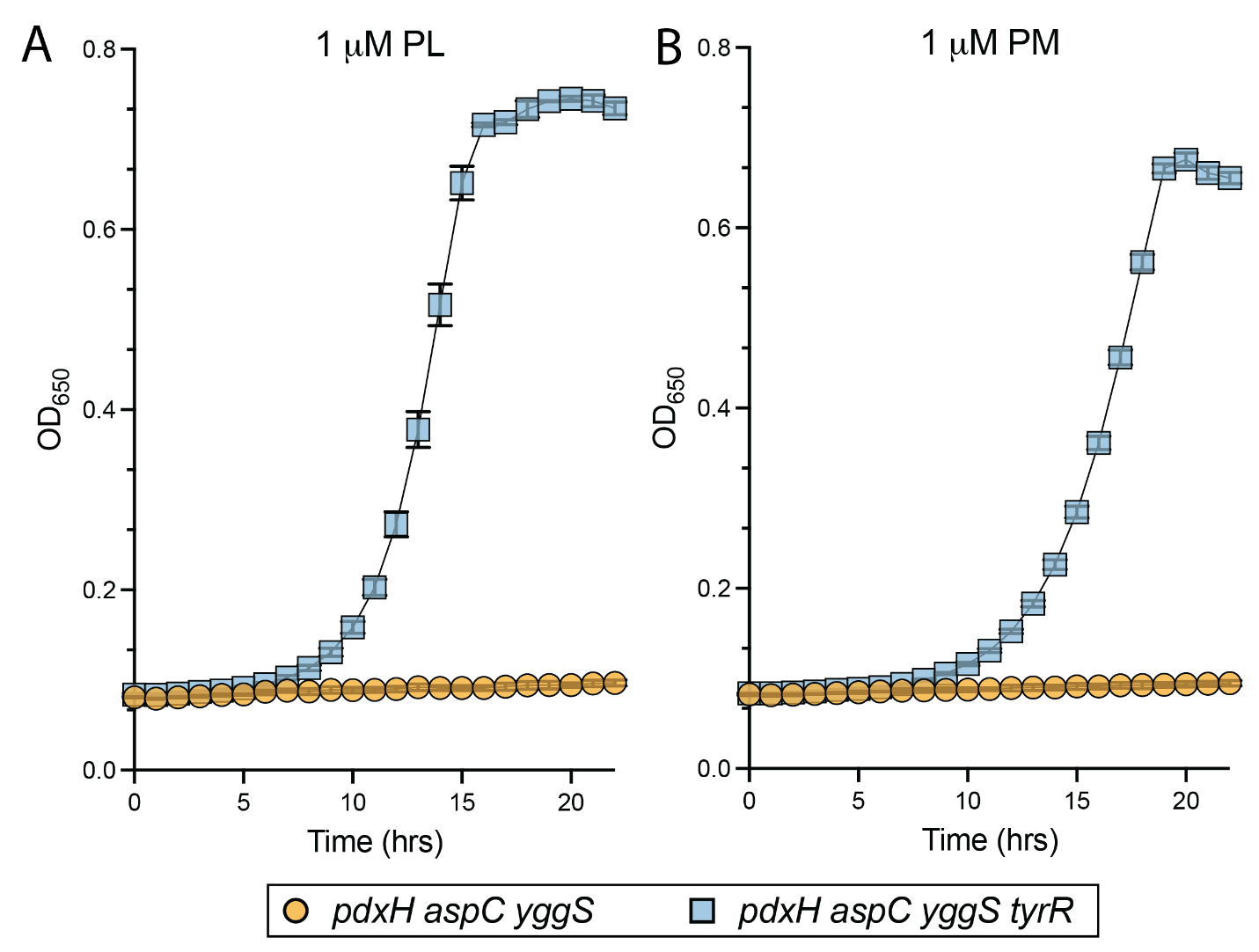
**

**Figure S4**. **Upregulation of *tyrB* rescues growth of *pdxH aspC yggS* strain.** Growth of *pdxH aspC yggS* (circles, DM16840) and *pdxH aspC yggS tyrR* (squares, DM18569) in minimal glycerol media supplemented with (A) 1 µM PL or (B) 1 µM PM. Error bars represent the standard deviation of three biological replicates.


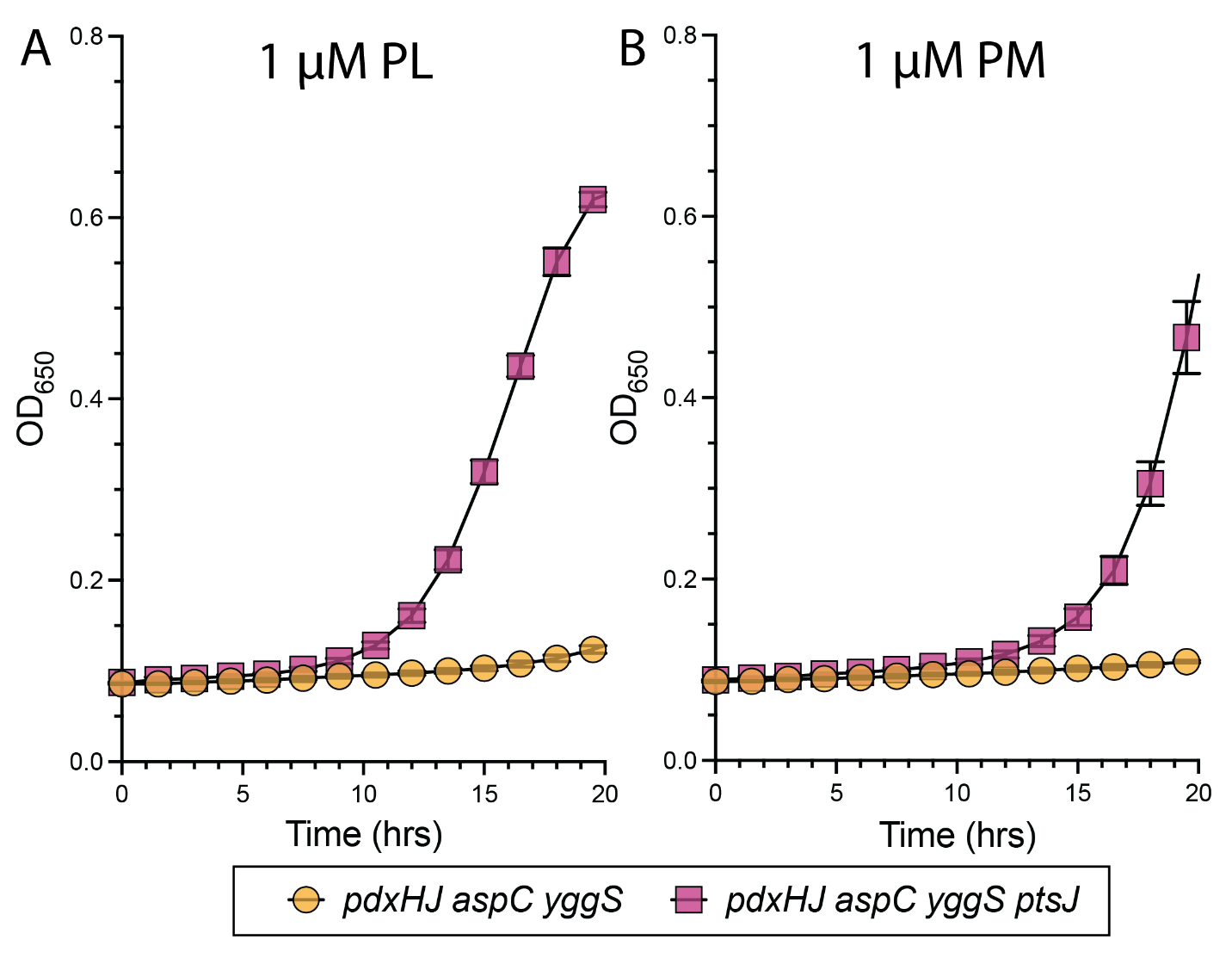


**Figure S5. Upregulation of PdxK allows growth of *pdxHJ aspC yggS* on low vitamer concentrations.** Growth of *pdxHJ aspC yggS* (circles, DM16705) and *pdxHJ aspC yggS ptsJ* (squares, DM18755) mutant strains on minimal media supplemented with (A) 1 μM PL or (B) 1 μM PM. Error bars represent the standard deviation of three biological replicates.


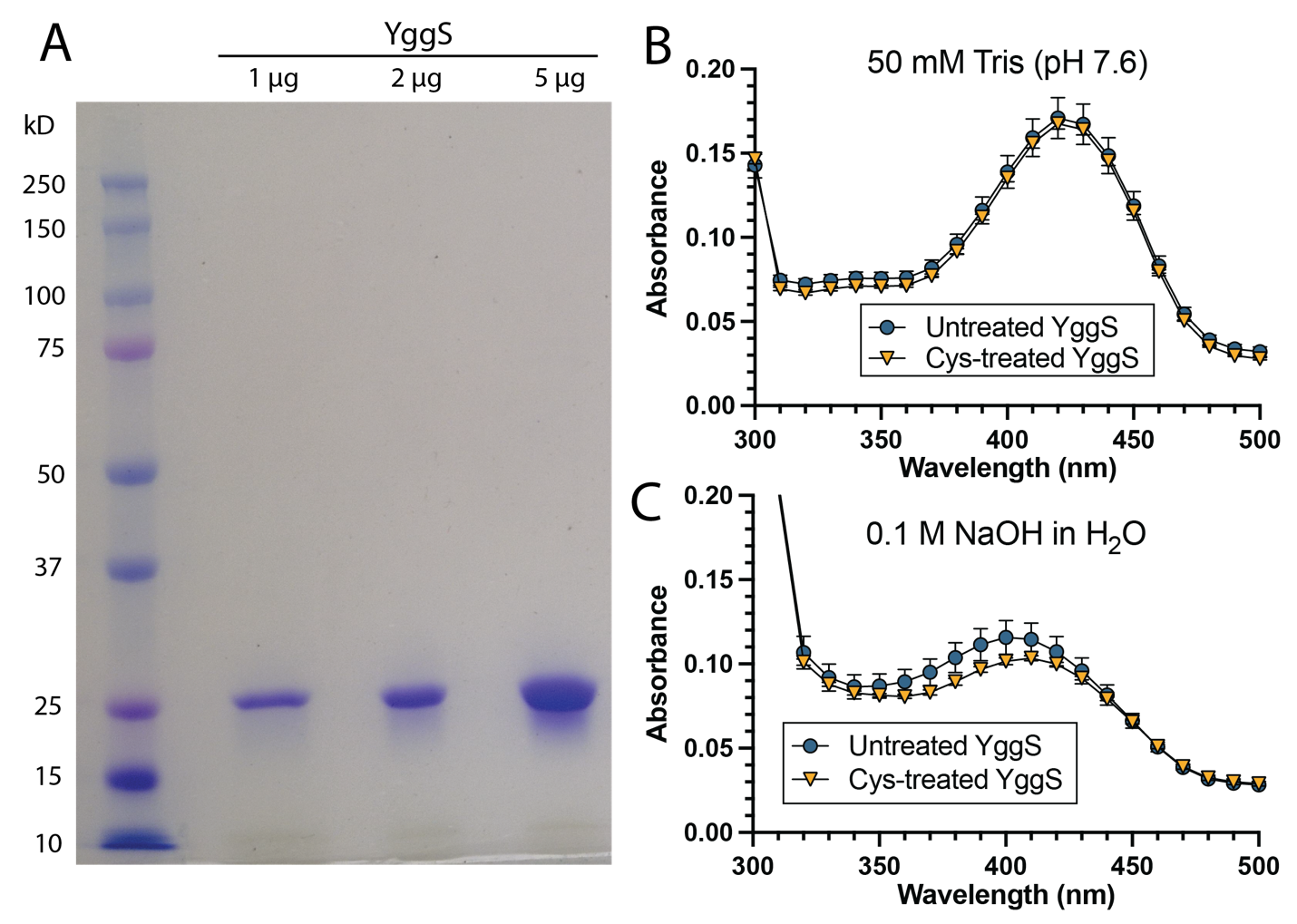


**Figure S6. Preparation of YggS protein. (**A) Purified YggS protein visualized by Coomaisee blue staining of a 10% SDS-PAGE gel. (B) UV spectra of untreated and cysteine-treated YggS (150 µM) in 50 mM Tris buffer (pH 7.6). Error bars represent standard deviation of three technical replicates. (C) UV spectra obtained after YggS protein (150 µM) was denatured in 0.1 M NaOH to release protein-bound PLP. Error bars represent standard deviation of three technical replicates.

REFERENCES

Datsenko, K.A., Wanner, B.L., 2000. One-step inactivation of chromosomal genes in Escherichia coli K-12 using PCR products. Proceedings of the National Academy of Sciences 97, 6640–6645. https://doi.org/10.1073/pnas.120163297

Galloway, N.R., Toutkoushian, H., Nune, M., Bose, N., Momany, C., 2013. Rapid Cloning For Protein Crystallography Using Type IIS Restriction Enzymes. Cryst. Growth Des. 13, 2833–2839. https://doi.org/10.1021/cg400171z

Way, J.C., Davis, M.A., Morisato, D., Roberts, D.E., Kleckner, N., 1984. New Tn10 derivatives for transposon mutagenesis and for construction of lacZ operon fusions by transposition. Gene 32, 369–379. https://doi.org/10.1016/0378-1119(84)90012-X
